# TRIM56 protects against nonalcoholic fatty liver disease by promoting the degradation of fatty acid synthase

**DOI:** 10.1172/JCI166149

**Published:** 2024-01-11

**Authors:** Suowen Xu, Xiumei Wu, Sichen Wang, Mengyun Xu, Tingyu Fang, Xiaoxuan Ma, Meijie Chen, Jiajun Fu, Juan Guo, Song Tian, Tian Tian, Xu Cheng, Hailong Yang, Junjie Zhou, Zhenya Wang, Yanjun Yin, Wen Xu, Fen Xu, Jinhua Yan, Zhihua Wang, Sihui Luo, Xiao-Jing Zhang, Yan-Xiao Ji, Jianping Weng

**Affiliations:** 1 Department of Endocrinology, Institute of Endocrine and Metabolic Diseases, The First Affiliated Hospital of USTC, Division of Life Sciences and Medicine, Clinical Research Hospital of the Chinese Academy of Sciences (Hefei), University of Science and Technology of China, Hefei, China.; 2 Department of Endocrinology, Guangdong Geriatrics Institute, Guangdong Provincial People’s Hospital, Guangdong Academy of Medical Sciences, Southern Medical University, Guangzhou, China.; 3 Department of Endocrinology and Metabolism, The Third Affiliated Hospital of Sun Yat-sen University, Guangzhou, China.; 4 School of Basic Medical Sciences, Wuhan University, Wuhan, China.; 5 State Key Laboratory of New Targets Discovery and Drug Development for Major Diseases, Gannan Innovation and Translational Medicine Research Institute and; 6 School of Medical Information Engineering, Gannan Medical University, Gannan Medical University, Ganzhou, China.; 7 College of Life Sciences, Wuhan University, Wuhan, China.; 8 Department of Cardiology, Renmin Hospital of Wuhan University, Wuhan, China.; 9 School of Pharmacy, Bengbu Medical College, Bengbu, China.

**Keywords:** Hepatology, Ubiquitin-proteosome system

## Abstract

Nonalcoholic fatty liver disease (NAFLD) encompasses a disease continuum from simple steatosis to nonalcoholic steatohepatitis (NASH). However, there are currently no approved pharmacotherapies for NAFLD, although several drugs are in advanced stages of clinical development. Because of the complex pathophysiology and heterogeneity of NAFLD, the identification of potential therapeutic targets is clinically important. Here, we demonstrated that tripartite motif 56 (TRIM56) protein abundance was markedly downregulated in the livers of individuals with NAFLD and of mice fed a high-fat diet. Hepatocyte-specific ablation of TRIM56 exacerbated the progression of NAFLD, while hepatic TRIM56 overexpression suppressed it. Integrative analyses of interactome and transcriptome profiling revealed a pivotal role of TRIM56 in lipid metabolism and identified the lipogenesis factor fatty acid synthase (FASN) as a direct binding partner of TRIM56. TRIM56 directly interacted with FASN and triggered its K48-linked ubiquitination–dependent degradation. Finally, using artificial intelligence–based virtual screening, we discovered an orally bioavailable small-molecule inhibitor of FASN (named FASstatin) that potentiates TRIM56-mediated FASN ubiquitination. Therapeutic administration of FASstatin improved NAFLD and NASH pathologies in mice with an optimal safety, tolerability, and pharmacokinetics profile. Our findings provide proof of concept that targeting the TRIM56/FASN axis in hepatocytes may offer potential therapeutic avenues to treat NAFLD.

## Abstract 2

The TRIM56/FASN axis regulates hepatocyte lipid accumulation in Nonalcoholic liver disease and FASN inhibition protects mice against HFD-induced hepatic steatosis.

## Graphical abstract

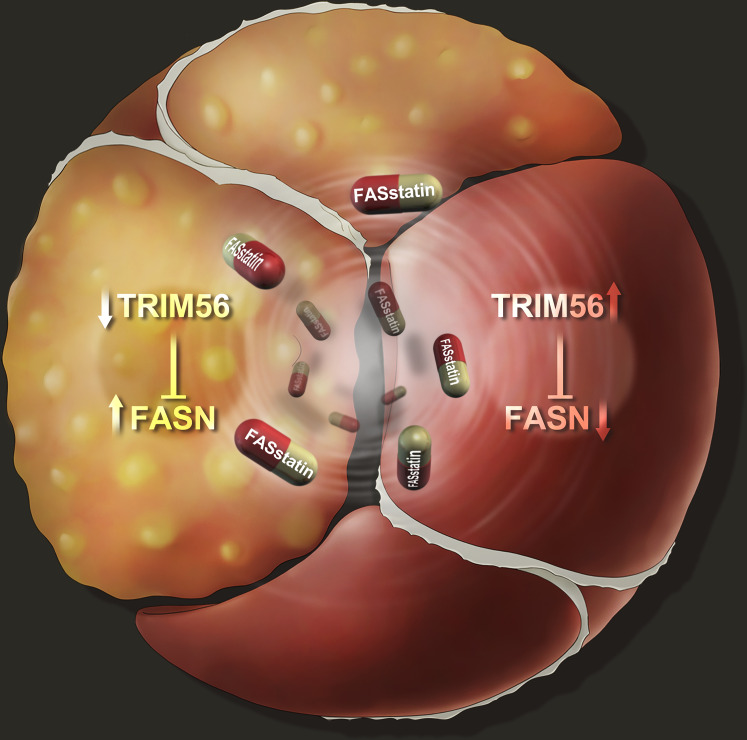

## Introduction

Nonalcoholic fatty liver disease (NAFLD) is the most prevalent metabolic dysfunction–associated steatotic liver disease due to the global obesity epidemic (1–3). The complex interplay between genetic, epigenetic, metabolic, dietary and environmental factors underlies the mechanistic basis of NAFLD (4). NAFLD remains silent and asymptomatic even until it progresses to nonalcoholic steatohepatitis (NASH), the advanced stage of NAFLD featuring hepatic steatosis, inflammation, fibrosis, and liver damage that could ultimately lead to life-threatening sequelae, including cirrhosis, hepatocellular carcinoma (HCC), and liver failure (5, 6). Currently, only bariatric-metabolic surgery, lifestyle interventions, and dietary alterations are recommended for the treatment of NAFLD (7). Although there are more than 70 candidate drugs in the therapeutic pipeline, no disease-modifying agents have been approved as effective pharmacotherapies against NAFLD and NASH (NAFLD/NASH) (5), highlighting the urgent medical need to identify target-based medical therapy for NAFLD/NASH.

Metabolic perturbations in NAFLD/NASH are driven by fatty acid synthesis pathways (8, 9). Increased fatty acid synthesis driven by nutrient surplus leads to lipotoxicity and metabolic stress and the resultant body weight gain and cardiometabolic risk factors associated with NAFLD/NASH (10). The ubiquitin/proteasome pathway is crucial for protein degradation and thus controls protein turnover and could potentially regulate diet-induced metabolic dysfunction. The extent of ubiquitination is determined by the concerted actions of ubiquitinases and deubiquitinases. In this regard, tripartite motif–containing (TRIM-containing) proteins comprise a large family of RING domain–containing E3 ubiquitin ligases that regulate inflammation, immunity, fibrosis, and cancer (11–14). However, only a few members of the TRIM family have been investigated in the context of metabolic disorders thus far.

In the present study, with the aim of discovering essential molecular factor(s) controlling NAFLD pathogenesis, we established a multilayer screening approach, ranging from transcriptomics analysis of human and mouse NAFLD samples to siRNA library screening followed by functional evaluation. We discovered that the TRIM family is a conserved protein family that plays important roles in hepatic steatosis and NAFLD development. Among the TRIM family members, we identified TRIM56 as an endogenous negative regulator of hepatic lipid accumulation by directly interacting with fatty acid synthase (FASN) and promoting its degradation. More important, by integrating artificial intelligence–assisted (AI-assisted) drug discovery, we identified a FASN inhibitor, FASstatin, that can promote TRIM56-mediated FASN protein degradation and suppress NAFLD in mice with a good efficacy and safety profile. The present study extends the recognized role of TRIM56 in antiviral innate immunity (15) and offers a potential pharmaceutical intervention strategy for NAFLD by targeting the TRIM56/FASN axis.

## Results

### Identification of TRIM56 as a key regulator of NAFLD.

**Figure 1:**
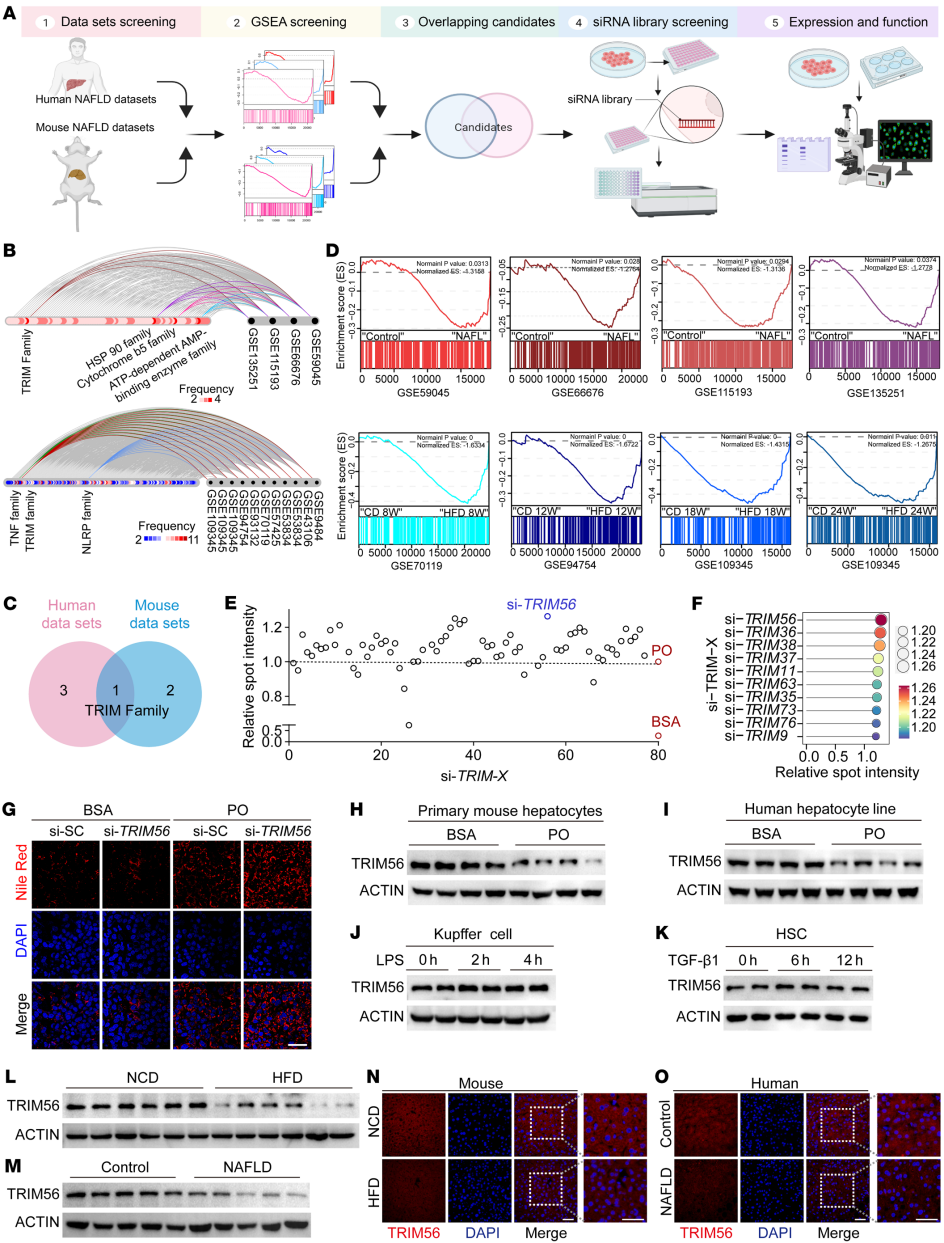
Identification of TRIM56 as the key regulator of NAFLD. (**A**) Workflow for the discovery of key regulator(s) of NAFLD. (**B**) Hive plot showing the top protein families associated with NAFLD in RNA-Seq data sets from human (upper panel) and mouse (lower panel) NAFLD data sets. (**C**) Venn diagram of panel **B**. (**D**) GSEA analysis of TRIM protein family members in individuals with NAFL (upper panel) and in mice with NAFLD after HFD feeding for different weeks (lower panel). ES, enrichment score; W, weeks. (**E**) Scatterplot of Nile Red–stained lipid droplet content in palmitic acid (0.5 mM) and oleic acid–challenged (1.0 mM) (PO-challenged) human hepatocytes after knockdown of TRIM family member proteins) (*n* = 4). Results for spot intensity relative to that of PO-challenged cells are presented. (**F**) Lollipop chart of top 10 hit using average fold change as the readout. (**G**) Representative images of Nile Red staining for TRIM56 knockdown in the presence or absence of PO (*n* = 3). (**H**) Western blotting of TRIM56 in mouse primary hepatocytes treated with BSA or PO for 18 hours (*n* = 4). (**I**) Western blotting of TRIM56 in HepG2 cells treated with BSA or PO for 18 hours (*n* = 3). (**J**) Western blotting of TRIM56 in mouse Kupffer cells that were treated with LPS (1 μg/mL) (*n* = 3). (**K**) Western blotting of TRIM56 in isolated primary mouse HSCs were treated with recombinant mouse TGF-β1 (10 ng/mL) for the indicated durations (*n* = 3). (**L**) Western blotting of TRIM56 in liver tissues from mice fed a normal chow diet (NCD) or a HFD for 24 weeks (*n* = 6). (**M**) Western blotting of TRIM56 in liver samples from individuals with NAFLD or control group (*n* = 5). (**N**) Representative immunofluorescence staining for TRIM56 (red) in liver tissues from HFD- and NCD-fed mice (*n* = 6). TRIM56 (red); DAPI (blue). Scale bars: 50 μm. (**O**) Representative immunofluorescence staining for TRIM56 (red) in liver tissues from individuals with NAFLD and healthy control individuals (*n* = 6). Scale bars: 50 μm.

To elucidate potential therapeutic targets of NAFLD, we first performed an integrative analysis of published mouse and human NAFLD data sets. In total, we included 4 human NAFLD published data sets and 11 mouse NAFLD data sets in our analysis. Candidate protein families were identified from the intersection of gene sets enriched in the NAFLD group in all human NAFLD databases and the gene sets enriched in the HFD group in all mouse NAFLD databases. Our analysis pinpoints TRIM protein family as a conserved family with altered gene expression in mouse and human NAFLD (Figure 1, A–D). To date, the role of the TRIM family of E3 ligases in hepatocyte lipid metabolism remains largely unexplored. Until now, over 70 TRIM family members have been identified. In order to further reveal the key TRIM family member in hepatic steatosis, we performed an unbiased siRNA library screening of 73 TRIM family members using Nile Red–based lipid accumulation in palmitic acid/oleic acid–challenged (PO-challenged) hepatocytes as the functional readout. We transfected hepatocytes with individual siRNAs at 50 nM for 48 hours in the presence of PO exposure before Nile Red staining by high-content imaging. Our screening data revealed that TRIM56 was a prominent suppressor of lipid accumulation compared with other TRIM members (Figure 1, E and F). Validation studies indicated that TRIM56 depletion by siRNA further aggravated PO-induced lipid accumulation (Figure 1G and Supplemental Figure 1; supplemental material available online with this article; https://doi.org/10.1172/JCI166149DS1).

We next explored the question of whether hepatocyte-derived TRIM56 plays an important role in hepatic steatosis and NAFLD pathogenesis. Since lipotoxicity driven by excessive free fatty acid accumulation is a key hallmark of NAFLD, we first evaluated protein expression of TRIM56 upon exposure to PO in primary mouse hepatocytes, as well as to LPS in Kupffer cells and TGF-β1 in hepatic stellate cells (HSCs). We observed that downregulation of TRIM56 protein occurred only in PO-challenged mouse and human hepatocytes (Figure 1, H and I), instead of LPS-challenged Kupffer cells (Figure 1J) or TGF-β1–challenged HSCs (Figure 1K). We then examined the effect of NAFLD on TRIM56 gene and protein expression in liver tissues. We observed that the protein expression level, rather than the gene expression level, of TRIM56 was markedly downregulated in mice fed a high-fat diet (HFD) as well as in individuals with NAFLD, as revealed by Western blot analysis and immunofluorescence staining (Figure 1, L–O, and Supplemental Figure 2, A–C). Further analysis revealed that PO challenge increased TRIM56 ubiquitination, and TRIM56 underwent self-ubiquitination as the E3-ligase catalytically inactive mutant of TRIM56 failed to cause ubiquitination of TRIM56 (Supplemental Figure 2, D and E). These data highlight a potential role of hepatocyte-derived TRIM56 in the progression of NAFLD. Altogether, these results indicate that the downregulation of TRIM56 was associated with NAFLD.

### TRIM56 blocks hepatocyte lipid accumulation.

**Figure 2:**
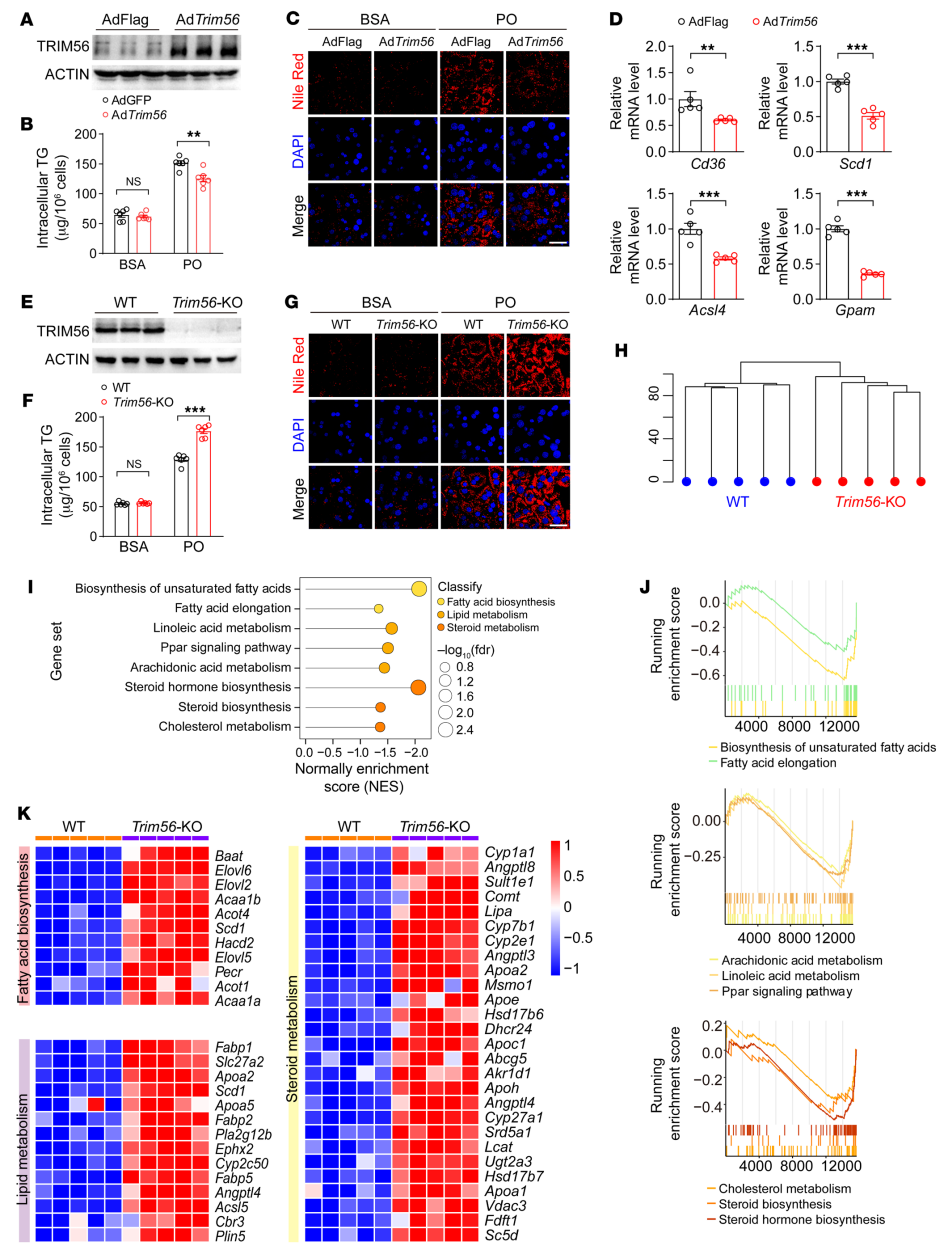
TRIM56 blocks hepatocyte lipid accumulation. (**A**) Primary mouse hepatocytes were infected with control adenovirus (AdFLAG) or *Trim56* adenovirus (Ad*Trim56*) for 24 hours before Western blotting was performed to determine the extent of TRIM56 overexpression (*n* = 3). (**B**) Intracellular TG levels were determined in primary mouse hepatocytes infected with AdFLAG or Ad*Trim56* in the presence of PO (*n* = 6). One-way ANOVA followed by Bonferroni’s post hoc test. (**C**) Primary mouse hepatocytes were infected with AdFLAG or Ad*Trim56* in the presence of PO. Nile Red staining was performed to visualize lipid droplet accumulation (*n* = 3). Scale bar: 50 μm. (**D**) Relative mRNA levels of genes related to fatty acid metabolism (*Cd36*, *Scd1*, *Acsl4*, *Gpam*) in the indicated groups in the presence of PO (*n* = 5). Mann-Whitney *U* test for *Cd36*; 2-tailed Student’s *t* test for *Scd1*, *Acsl4*, and *Gpam*. (**E**) TRIM56 protein expression in hepatocytes isolated from WT and *Trim56* global-KO (*Trim56*-KO) mice (*n* = 3). (**F**) Primary hepatocytes were isolated from WT and *Trim56*-KO mice. Intracellular TG levels were determined in PO-challenged primary hepatocytes (*n* = 6). One-way ANOVA followed by Tamhane’s T2 analysis. (**G**) WT or *Trim56*-KO hepatocytes were treated with PO for 18 hours before Nile Red staining was performed to visualize lipid droplet accumulation (*n* = 3). Scale bar: 50 μm. (**H**) Cluster profile of WT and *Trim56*-KO hepatocytes treated with PO (*n* = 5). (**I**) Enriched pathways in *Trim56*-KO hepatocytes versus WT hepatocytes treated with PO. (**J**) GSEA analysis of pathways displayed in **I**. (**K**) RNA-Seq heatmap analysis of differentially expressed genes between WT and *Trim56*-KO hepatocytes treated with PO. Differentially expressed genes in pathways of lipid metabolism, fatty acid metabolism, and steroid metabolism are highlighted. **P < 0.01 and ***P < 0.001 (**B**, **D**, and **F**).

On the basis of the observations that TRIM56 protein expression was decreased in NAFLD mice, humans, and PO-challenged hepatocytes, we hypothesized that TRIM56 could potentially confer protection against NAFLD. To determine the precise role of *Trim56* in lipid accumulation in vitro, we applied Nile Red staining as a proxy for cytosolic lipid droplet accumulation. We first explored the possibility that TRIM56 gain of function by adenovirus-mediated overexpression could attenuate lipid accumulation in hepatocytes. We observed that *Trim56* overexpression dampened lipid droplet accumulation and mitigated intracellular triglyceride (TG) accumulation in hepatocytes stimulated with PO (Figure 2, A–C). Consistently, the expression of genes associated with fatty acid metabolism was attenuated by *Trim56* overexpression (Figure 2D). In contrast, *Trim56*-deficient hepatocytes accumulated more lipid droplets upon PO stimulation with a paralleled increase in intracellular TG measurements (Figure 2, E–G). To dissect the role of *Trim56* in lipid metabolism, we performed RNA-Seq analysis on WT and *Trim56*-deficient hepatocytes upon exposure to PO. Our transcriptomic profiling data revealed that TRIM56 ablation led to the upregulation of pathways enriched in fatty acid biosynthesis, lipid metabolism, and steroid metabolism (Figure 2, H–K). Collectively, these results suggest a potential role for TRIM56 in protection against NAFLD.

### Hepatocyte TRIM56 protects against HFD-induced hepatic steatosis.

**Figure 3:**
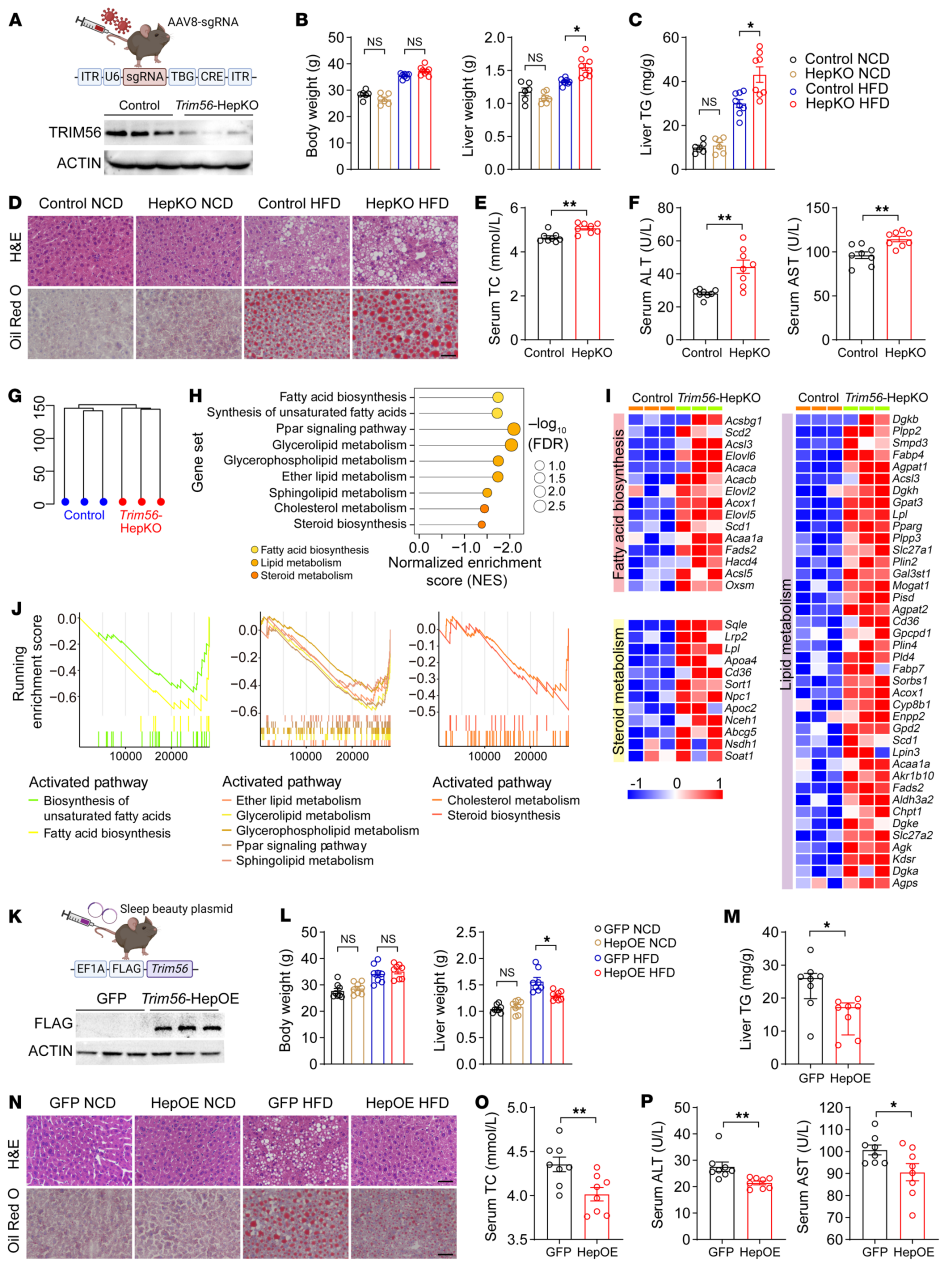
Hepatocyte TRIM56 protects against HFD-induced hepatic steatosis. (**A**) Scheme for the generation of hepatocyte-specific Trim56-KO mice (Trim56-HepKO) and western blot validation (*n* = 3). (**B**) Body weight and liver weight of WT and Trim56-HepKO after 16 weeks of HFD feeding (*n* = 6 for NCD; *n* = 8 for HFD). One-way ANOVA followed by Bonferroni’s post hoc test for body weight and Tamhane’s T2 analysis for liver weight. (**C**) Liver TG content in WT and Trim56-HepKO. after 16 weeks of HFD feeding (*n* = 6 for NCD; *n* = 8 for HFD) for **B** and **C**. (**D**) Representative H&E and Oil Red O staining of liver sections from mice indicated groups of the indicated groups (*n* = 4). Scale bars: 50 μm. (**E**) Serum TC content of WT and Trim56-HepKO mice after 16 weeks of HFD feeding (*n* = 8). Two-tailed Student’s *t* test. (**F**) Serum ALT and AST levels of the mice after 16 weeks of HFD feeding (*n* = 8). Two-tailed Student’s *t* test. (**G**) Cluster profile of WT and Trim56-HepKO mice fed a HFD for 16 weeks. (**H**) Enriched pathway analysis of liver tissues from Trim56-HepKO mice versus control mice (*n* = 3). (**I**) Heatmap illustrating overrepresented pathways of fatty acid biosynthesis, lipid metabolism, and steroid metabolism genes in liver tissues from Trim56-HepKO mice (*n* = 3). (**J**) GSEA showing overrepresentedthat pathways of fatty acid biosynthesis, lipid metabolism, and steroid metabolism genes were overrepresented in liver tissues from Trim56-HepKO mice (*n* = 3). (**K**) Scheme for the generation of hepatocyte-specific Trim56-overexpressing mice (Trim56-HepOE) and using Trim56–sleeping beauty plasmid injection. Control mice receive an injection of empty vector. Successful deletion of TRIM56 protein in the liver was verified by Western blotting validation (*n* = 3). (**L**) Body weight and liver weight of WT (GFP) and Trim56-HepOE mice after 16 weeks of NCD or HFD feeding (*n* = 8). One-way ANOVA followed by Bonferroni’s post hoc test for body weight and Tamhane’s T2 analysis for liver weight. (**M**) Liver TG content in WT and Trim56-HepOE mice after 16 weeks of HFD feeding (*n* = 8). Mann-Whitney *U* test. (**N**) Representative H&E and Oil Red O staining of liver sections from mice in the indicated groups (*n* = 3). Scale bars: 50 μm. (**O**) Serum TC content of WT and Trim56-HepOE mice after 16 weeks of HFD feeding (*n* = 8). Two-tailed Student’s *t* test. (**P**) Serum ALT and AST content levels in WT and Trim56-HepOE mice after 16 weeks of HFD feeding (*n* = 8). Two-tailed Student’s *t* test. *P < 0.05 and **P < 0.01.

To investigate the effect of TRIM56 in hepatocytes on NAFLD pathogenesis in vivo, we generated hepatocyte-specific *Trim56*-KO mice by AAV8-*Trim56*-sgRNA-TBG-Cre injection. We observed significantly decreased TRIM56 protein levels in the livers of *Trim56* conditional-KO (*Trim56*-HepKO) mice (Figure 3A). We then evaluated the effect of hepatocyte-specific *Trim56* ablation on HFD-accelerated NAFLD development in mice. We observed that liver weights and hepatic TG levels were increased in the *Trim56*-HepKO mice (Figure 3, B and C). Similarly, we observed increased hepatic steatosis and lipid droplet accumulation in *Trim56*-HepKO mice (Figure 3D). In addition, circulating levels of total cholesterol (TC), ALT, and AST were also increased in *Trim56*-HepKO mice (Figure 3, E and F). To dissect the molecular mechanism of *Trim56* in NAFLD, we performed RNA-Seq analysis in WT and *Trim56*-HepKO mouse liver. Our data revealed that pathways of fatty acid biosynthesis, lipid metabolism, and steroid metabolism were overrepresented in *Trim56*-HepKO mouse liver tissues (Figure 3, G–J).

Given the effect of *Trim56* deletion on augmented lipid metabolism–associated signaling pathways in hepatocytes, we next investigated the effect of hepatocyte-specific *Trim56*- overexpression on NAFLD pathogenesis. We generated *Trim56* hepatocyte–specific–overexpressing mice (*Trim56*-HepOE) using the Sleeping Beauty Transpose system (16) and subjected them to a HFD challenge. We observed that TRIM56 was successfully overexpressed and that *Trim56*-HepOE mice showed protection against NAFLD evidenced by decreased liver weight, hepatic TG levels, and hepatic lipid accumulation (Figure 3, K–N). Serum levels of TC, ALT, and AST were also lowered in *Trim56*-HepOE mice (Figure 3, O and P).

### Identification of FASN as a TRIM56 binding partner.

**Figure 4:**
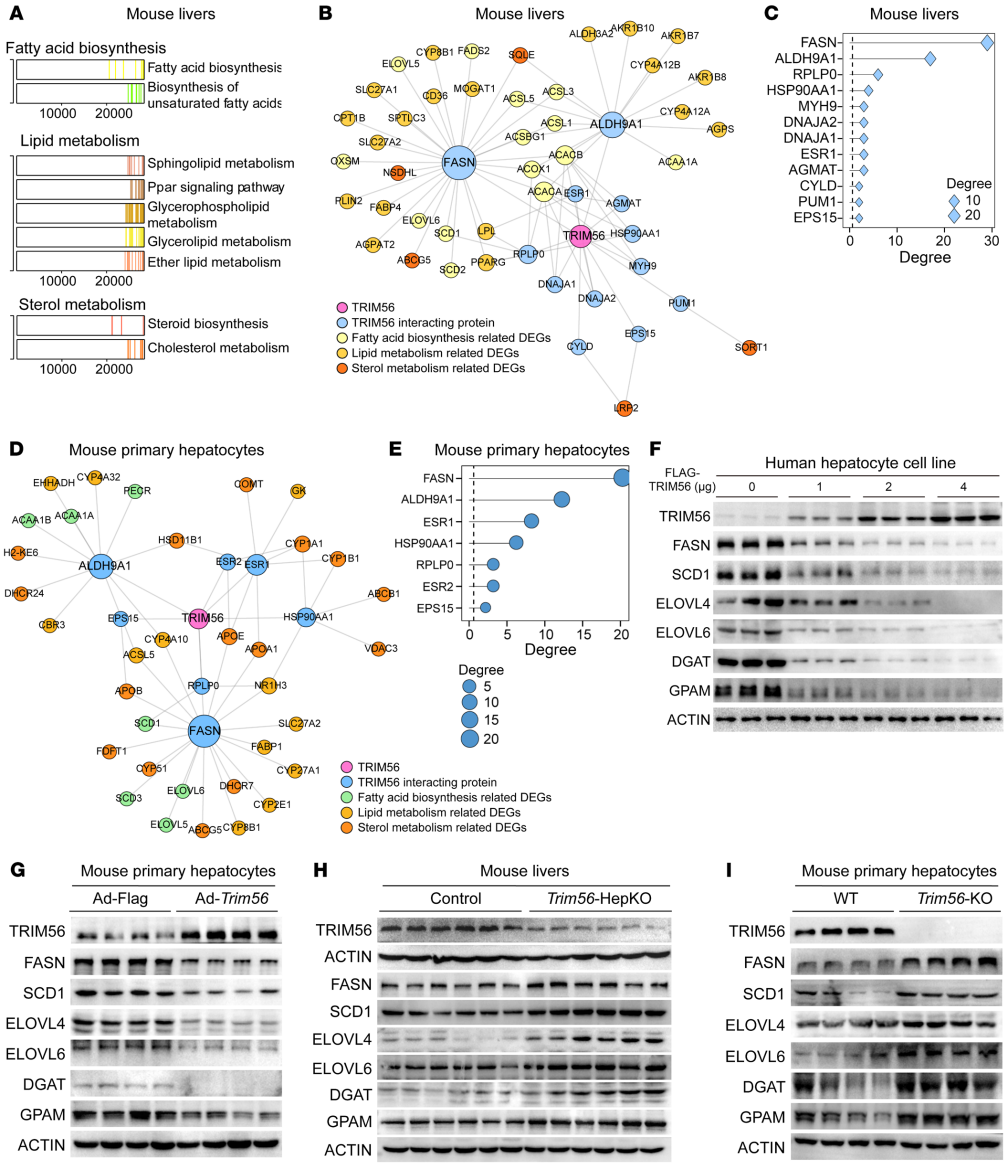
Identification of FASN as a TRIM56 binding partner. (**A**) Enriched pathway analysis in *Trim56*-HepKO mouse liver tissues as revealed by RNA-Seq. (**B**) Integrated analysis of TRIM56 interactomics and RNA-Seq of the samples described in **A**. (**C**) Ranking of candidate proteins interacting with TRIM56 in mouse liver tissues. (**D**) Integrated analysis of TRIM56 interactomics and RNA-seq of samples from *Trim56*-KO hepatocytes. (**E**) Ranking of candidate proteins interacting with TRIM56 in mouse hepatocytes. (**F**) HepG2 cells were transfected with empty vector (FLAG) or increasing amounts of FLAG-TRIM56 (0, 1, 2, and 4 μg) in the presence of PO before whole-cell lysate was collected for Western blotting to determine protein expression levels in the lipogenesis pathway (SCD1, ELOVL4, ELOVL6, DGAT, and GPAM) (*n* = 3). (**G**) Primary mouse hepatocytes were infected with AdFLAG or AdFLAG-*Trim56* in the presence of PO before whole-cell lysate was collected for Western blotting to determine the expression of proteins in the lipogenesis pathway (*n* = 4). (**H**) Expression of FASN and downstream proteins associated with the lipogenesis pathway in WT and *Trim56*-HepKO mouse liver tissues under HFD conditions (*n* = 6). (**I**) Expression of FASN and downstream lipogenesis-related proteins in WT and *Trim56*-KO mouse hepatocytes treated with PO (*n* = 4).

To gain insight into the possible mechanism of TRIM56 in regulating NAFLD, we first performed an integrative analysis of reported TRIM56 interactomics (BioGrid) and transcriptomic profiling in *Trim56*-HepKO liver tissues, which yielded FASN as the top hit based on integrated score ranking (Figure 4, A–C). Integrative analysis of reported TRIM56 interactomics and transcriptomic profiling in *Trim56*-KO mouse hepatocytes yielded similar results (Figure 4, D and E). By Western blot analysis, we demonstrated that FASN protein expression was decreased in primary mouse hepatocytes as well as human hepatocyte lines overexpressing TRIM56 (Figure 4, F and G). In contrast, increased expression of FASN as well as of proteins in downstream lipogenesis cascades in the pathogenic process of fatty acid metabolism was observed in both liver tissues from *Trim56*-HepKO mice as well as in primary hepatocytes obtained from *Trim56*-KO mice (Figure 4, H and I), compared with respective controls.

### TRIM56 interacts with FASN and promotes its degradation.

**Figure 5:**
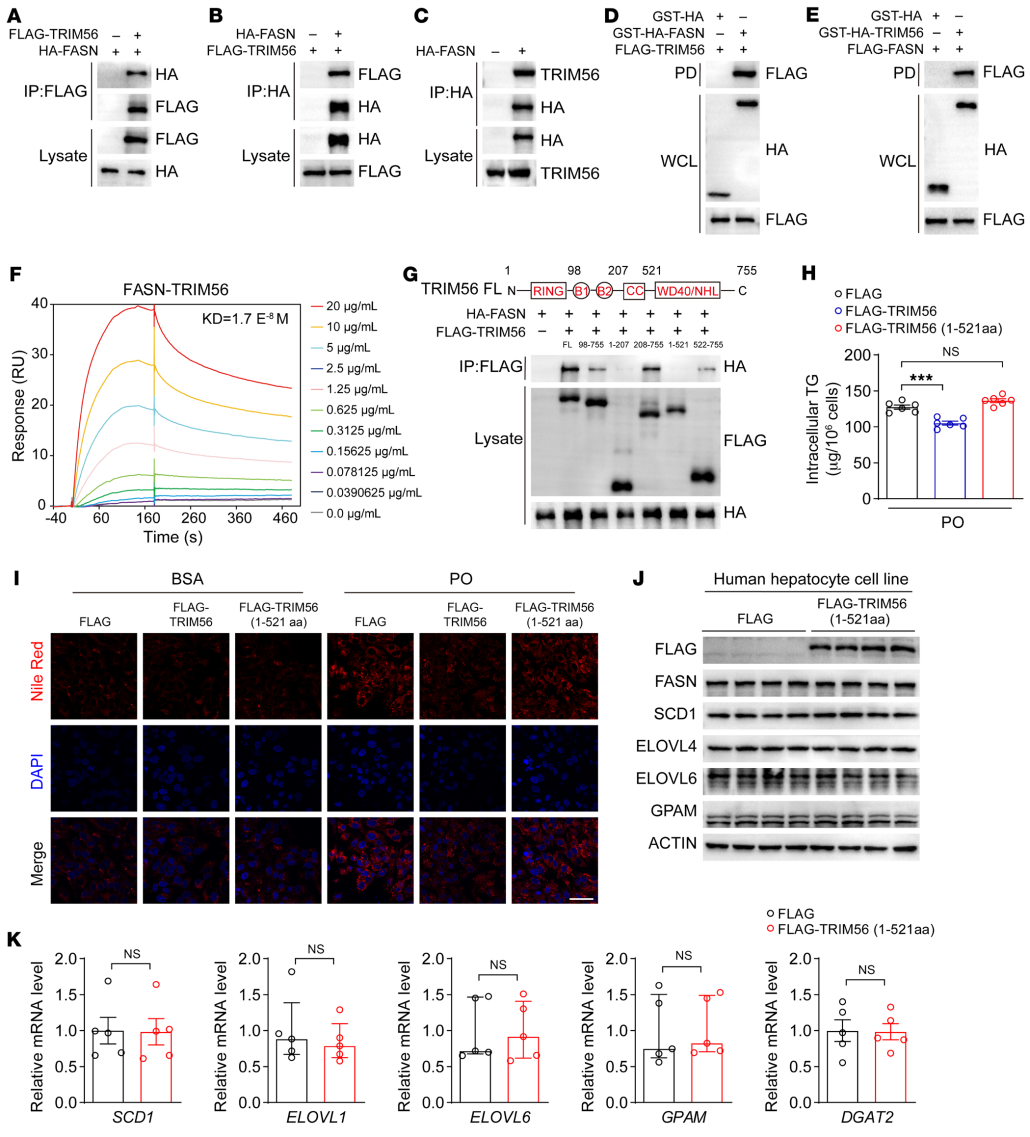
TRIM56 interacts with FASN. (**A** and **B**) Interaction of FLAG-TRIM56 with HA-FASN in HEK293T cells as demonstrated by IP (*n* = 3). (**C**) HA-FASN interacted with endogenous TRIM56 in HEK293T (*n* = 3). (**D** and **E**) GST-pulldown assay confirmed the interaction between TRIM56 and FASN in HEK293T cells (*n* = 3). (**F**) FASN-TRIM56 direct interaction as determined by SPR analysis. (**G**) Molecular characterization of FASN interaction with different truncated fragments of TRIM56 in HEK293T cells (*n* = 3). (**H**) Determination of intracellular TG content in HepG2 cells transfected with empty vector (FLAG) or FLAG -TRIM56 or the FLAG-TRIM56 (1–521 aa) fragment in the presence of PO (*n* = 6). ***P < 0.001, by 1-way ANOVA followed by Bonferroni’s post hoc test. (**I**) HepG2 were transfected with empty vector or FLAG-TRIM56, or FLAG-TRIM56 (1–521 aa) fragment in the presence or absence of PO before Nile Red staining (*n* = 3). Scale bars: 50 μm. (**J**) HepG2 cells were transfected with empty vector (FLAG) or FLAG-TRIM56 (1–521 aa) in the presence of PO before whole-cell lysate was collected for Western blotting to determine the expression of proteins related to fatty acid metabolism (*n* = 3). (**K**) Expression of the indicated genes in HepG2 cells transfected with empty vector (FLAG) or FLAG-TRIM56 (1–521 aa) in the presence of PO (*n* = 5). NS, by 2-tailed Student’s *t* test for *SCD1* and *DGAT2* and Mann-Whitney *U* test for *ELOVL1*, *ELOVL6*, and *GPAM*.

Given the above observations, it is plausible that TRIM56 interacts with FASN and promotes its degradation. We thus characterized the interaction between TRIM56 and FASN. By co-IP and GST pulldown assays, we found that TRIM56 interacted with FASN (Figure 5, A–E). Surface plasmon resonance (SPR) analysis further supported the finding that TRIM56 directly interacted with FASN (Figure 5F). Structurally, TRIM56 consists of a highly conserved RING domain, 2 B-boxes, and a coiled-coil (CC) domain, as well as a C-terminal domain (CTD) that confers function specificity. We then mapped the interaction domain of TRIM56 using different versions of truncated TRIM56 fragments and confirmed that aa 522–755 of TRIM56 were essential for the interaction between TRIM56 and FASN, as demonstrated by the loss of the interaction between TRIM56 (1–521 aa) and FASN (Figure 5G). Of functional relevance, overexpression of full-length TRIM56, but not TRIM56 (1–521 aa), the non-FASN-interacting TRIM56 fragment, reduced intracellular TG accumulation as well as lipid accumulation in hepatocytes (Figure 5, H and I). Furthermore, we show that TRIM56 (1–521 aa) overexpression did not lead to the downregulation of FASN or its downstream proteins associated with fatty acid metabolism. Furthermore, TRIM56 (1–521 aa) overexpression also had no effect on the expression of lipogenesis-related proteins or genes (Figure 5, J and K).

**Figure 6:**
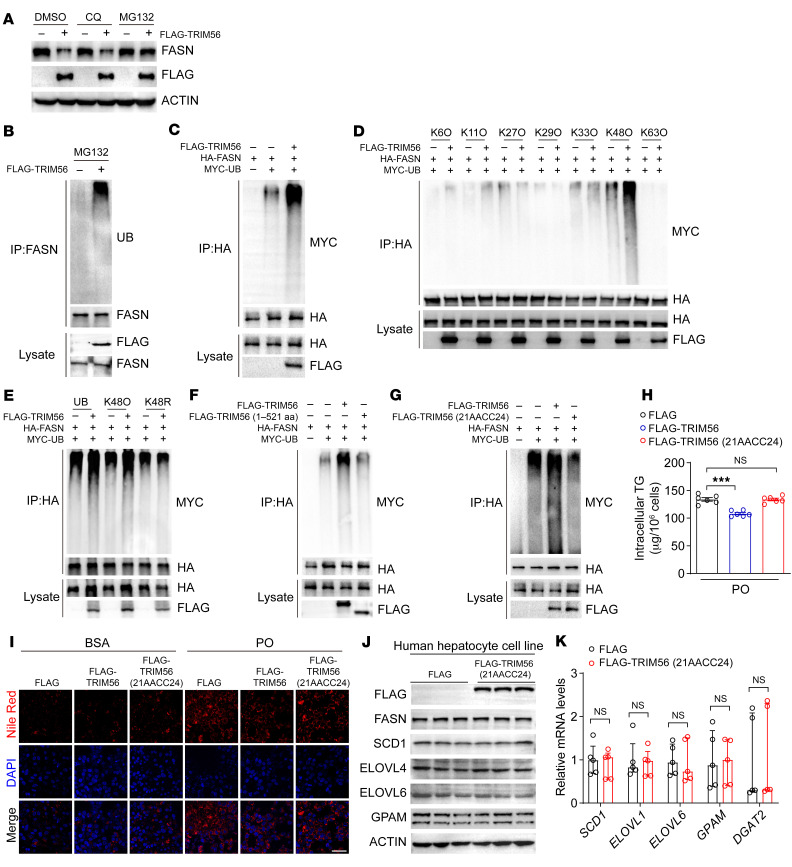
TRIM56 promotes FASN degradation. (**A**) HepG2 cells were transfected with empty vector or FLAG-TRIM56 in the presence of indicated compounds. FASN protein expression was determined (*n* = 3). (**B**) HEK293T cells were transfected with empty vector or FLAG-TRIM56 in the presence of MG132 before IP was performed (*n* = 3). (**C**) HEK293T cells were transfected with HA-FASN with or without FLAG-TRIM56 and MYC-ubiquitin before IP was performed (*n* = 3). (**D**) HEK293T cells were transfected with HA-FASN in the presence or absence of FLAG-TRIM56 and MYC tagged site-specific ubiquitin mutants. Then, IP was performed (*n* = 3). (**E**) HEK293T cells were transfected with HA-FASN with or without FLAG-TRIM56 and MYC-tagged active K48–linked ubiquitin (K48O) or inactive K48–linked ubiquitin (K48R) mutant. Then, IP was performed (*n* = 3). (**F**) HEK293T cells were transfected with HA-FASN in the presence or absence of FLAG-TRIM56 (full-length), FLAG-TRIM56 (1–521 aa), and MYC-ubiquitin (UB). Then, IP was performed (*n* = 3). (**G**) HEK293T cells were transfected with HA-FASN with or without FLAG-TRIM56, E3 ligase–defective mutant FLAG-TRIM56 (21AACC24), and MYC-UB. Then, IP was performed (*n* = 3). (**H**) Intracellular TG content (*n* = 6). ***P < 0.001, by 1-way ANOVA followed by Bonferroni’s post hoc test. (**I**) HepG2 cells were transfected with empty vector (FLAG) or FLAG-TRIM56, or FLAG-TRIM56 mutant (21AACC24) with or without PO before Nile Red staining (*n* = 3). Scale bar: 50 μm. (**J**) HepG2 cells were transfected with empty vector (FLAG) or FLAG-TRIM56 mutant (21AACC24) to determine the expression of the indicated proteins (*n* = 3). (**K**) Expression of the indicated genes in HepG2 transfected with empty vector (FLAG) or FLAG-TRIM56 mutant (21AACC24) in the presence of PO (*n* = 5). Two-tailed Student’s *t* test for *SCD1* and *GPAM*; Mann-Whitney *U* test for *ELOVL1*, *ELOVL6*, and *DGAT2*.

To further address the molecular mechanisms underlying TRIM56-mediated FASN degradation, we overexpressed FLAG-TRIM56 and treated hepatocytes with chloroquine (CQ), a pharmacological inhibitor of lysosome-dependent protein degradation, or MG132, an inhibitor of proteasome-dependent protein degradation. The Western blot analysis results showed that TRIM56 overexpression–induced FASN downregulation was rescued by MG132 rather than CQ, suggesting that proteasome-dependent degradation of FASN may play a major role (Figure 6A). Compared with cells treated with empty vector, overexpression of TRIM56 led to enhanced FASN polyubiquitination (Figure 6, B and C). Given that multiple sites existing in ubiquitin give rise to substrate ubiquitination, we examined the specific site of ubiquitination involved. We found that K48-linked polyubiquitination played a major role in the polyubiquitination of FASN (Figure 6D), which was further corroborated by the evidence showing increased FASN ubiquitination in K48O-overexpressing, but not K48R-overexpressing, cells (Figure 6E). However, deletion of the CTD of TRIM56 abrogated the TRIM56-FASN interaction, suggesting that CTD (522–755 aa) of TRIM56 interacted with FASN. Overexpression of the TRIM56 (1–521 aa) fragment (which is unable to bind with FASN) failed to increase FASN polyubiquitination, suggesting that aa position 522–575 is critical for TRIM56-FASN interaction and FASN protein instability (Figure 6F). More important, we demonstrated that, compared with the WT TRIM56, TRIM56 (21AACC24) (an E3 ubiquitin ligase activity–defective mutant) (15) failed to increase FASN protein ubiquitination (Figure 6G). Consistent with this observation, WT TRIM56, but not the TRIM56 (21AACC24) mutant, decreased PO-induced lipid accumulation and TG elevation (Figure 6, H and I). TRIM56 (21AACC24) did not affect lipogenesis-related gene or protein expression (Figure 6, J and K). Altogether, these lines of evidence demonstrate that TRIM56 kept hepatocyte steatosis in check by interacting with FASN and conjugated K48-linked ubiquitination chains to promotes the degradation of FASN.

### FASN inhibition blocks the effect of Trim56 ablation on lipid accumulation.

**Figure 7:**
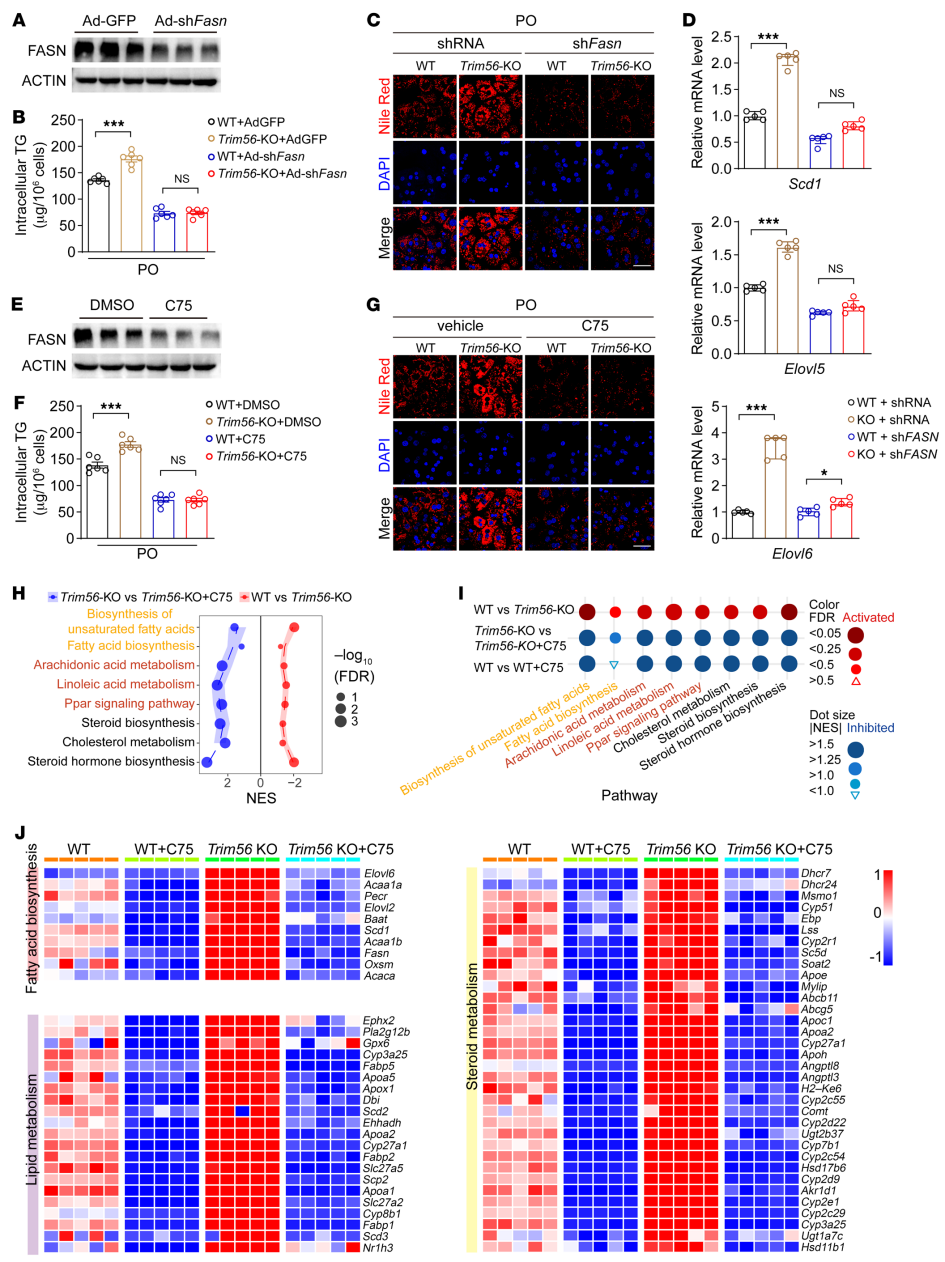
FASN inhibition blocks the effect of *Trim56* ablation on lipid accumulation. (**A**) Silencing efficiency of adenovirus vector encoding an shRNA against *Fasn* (Ad-sh*Fasn*) in primary mouse hepatocytes. Hepatocytes were infected with control adenovirus (Ad-GFP) or Ad-sh*Fasn* (MOI = 2) for 24 hours before whole-cell lysate was collected for Western blotting (*n* = 3). (**B**) Primary mouse hepatocytes were isolated from WT and *Trim56*-KO mice. Then, hepatocytes were infected with Ad-GFP or Ad-sh*Fasn* in the presence of PO for 18 hours before intracellular TG levels were determined (*n* = 6). One-way ANOVA followed by Bonferroni’s post hoc test. (**C**) Nile Red staining of WT and *Trim56*-KO hepatocytes treated as described in **B** (*n* = 3.). Scale bar: 50 μm. (**D**) mRNA expression of *Scd1*, *Elovl5*, and *Elovl6* in hepatocytes from the indicated groups (*n* = 5). One-way ANOVA followed by Bonferroni’s post hoc test for *Elovl5*; Kruskal-Wallis test for *Scd1* and *Elovl6*. (**E**) Effect of the FASN inhibitor C75 on FASN protein expression. Primary mouse hepatocytes were treated with vehicle (0.1%DMSO) or C75 (a pharmacological inhibitor of FASN, 20 μM) for 24 hours (*n* = 3). (**F**) Primary mouse hepatocytes were isolated from WT and *Trim56*-KO mice. Then, hepatocytes were treated with vehicle or C75 (20 μM) in the presence of PO before intracellular TG analysis (*n* = 6). One-way ANOVA followed by Bonferroni’s post hoc test. (**G**) Nile Red staining was performed on hepatocytes treated as described in **F** (*n* = 3). Scale bar: 50 μm. (**H**) Enriched pathway analysis in WT and *Trim56*-KO hepatocytes treated with vehicle control or C75 (20 μM) in the presence of PO (*n* = 5). (**I**) Dot analysis of the enriched pathways identified in **H** (*n* = 5). (**J**) Heatmap analysis revealed the expression profile of genes involved in lipid metabolism, fatty acid biosynthesis, and steroid metabolism in WT and *Trim56*-KO hepatocytes treated with vehicle or C75 (20 μM) (*n* = 5). *P < 0.05 and ***P < 0.001 (**B**, **D**, and **F**).

Given that FASN is required for the de novo lipogenesis (DNL) pathway, it remains to be elucidated whether FASN upregulation contributes to the altered lipid metabolism in *Trim56*-KO hepatocytes. We therefore asked whether decreasing FASN expression by shRNA-mediated knockdown or pharmacological inhibition of FASN activity by treatment with compound C75 could rescue the phenotype of *Trim56*-deficient hepatocytes upon metabolic challenge. We observed that sh*Fasn* significantly reduced FASN protein abundance (Figure 7A) and diminished TG accumulation in *Trim56*-KO hepatocytes (Figure 7B). FASN depletion also reversed the effect of *Trim56* deficiency on lipid droplet accumulation (Figure 7C) as well as the expression of genes involved in lipogenesis, fatty acid transport, and elongation (such as *Scd1*, *Elovl5*, and *Elovl6*) (Figure 7D). Likewise, pharmacological inhibition of FASN by compound C75 (17) also decreased FASN protein expression and reversed the effect of *Trim56* deficiency on lipid droplet formation and TG accumulation (Figure 7, E–G). We further performed RNA-Seq analysis of primary hepatocytes isolated from WT and *Trim56*-KO mice treated with or without C75. Our data vividly demonstrated upregulation of enriched pathways in fatty acid biosynthesis, lipid metabolism, and steroid metabolism. However, treatment with C75 nullified the deleterious effects of *Trim56* deficiency by reversing the activation of these pathways as well as the expression of the deregulated genes (Figure 7, H–J). These results indicate that direct interaction between TRIM56 and FASN was required for hepatic lipid accumulation and that the TRIM56/FASN axis may serve to integrate critical mechanistic nodes linking metabolic stress and NAFLD development.

### AI-based compound screening identifies FASstatin as an inhibitor of FASN.

**Figure 8:**
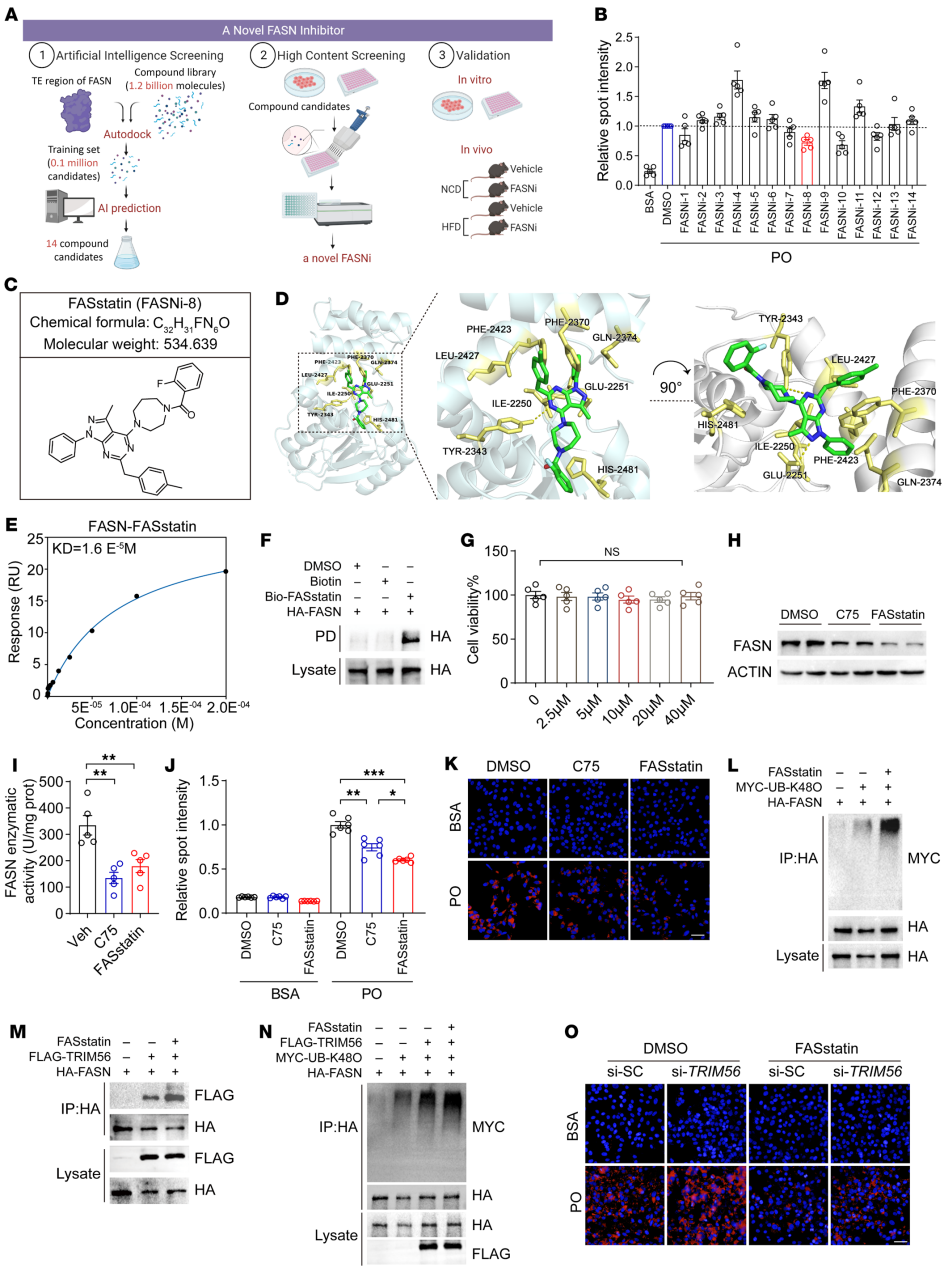
AI-based compound screening identifies FASstatin as an inhibitor of FASN. (**A**) High-throughput virtual screening workflow for lead compound identification and pharmacological validation. (**B**) Validation of top 14 hit FASNi by BODIPY staining in a PO-challenged human hepatocyte line (*n* = 5). (**C**) Chemical structure of the hit compound FASstatin (FASNi-8). (**D**) Molecular docking of FASstatin to the thioesterase domain of FASN. (**E**) Interaction of FASN and FASstatin in a SPR assay. (**F**) Biotin-labeled FASstatin interacted with FASN in a streptavidin bead–pulldown (PD) assay (*n* = 3). (**G**) Cell viability assay of HepG2 cells treated with increasing doses of FASstatin (*n* = 5). One-way ANOVA. (**H**) Effects of FASstatin (20 μM) and C75 (20 μM) on FASN protein expression in human hepatocyte lines (*n* = 3). (**I**) Effect of FASstatin (20 μM) and C75 (20 μM) on FASN enzyme activity (*n* = 5). One-way ANOVA followed by Bonferroni’s post hoc test. prot, protein; Veh, vehicle. (**J**) Effects of FASstatin (20 μM) and C75 (20 μM) on PO-induced lipid accumulation in Huh7 cells (*n* = 6). One-way ANOVA followed by Tamhane’s T2 analysis. (**K**) Representative immunofluorescence images of Nile Red staining of Huh7 cells treated as described in **H** (*n* = 6). Scale bar: 50 μm. (**L**) Effect of FASstatin on FASN ubiquitination at Lys 48 in human hepatocyte line (*n* = 3). (**M**) FASstatin potentiated TRIM56-FASN interaction in HEK293T cells (*n* = 3). (**N**) Effect of FASstatin on TRIM56-mediated, K48-linked ubiquitination of FASN in human hepatocyte line (*n* = 3). (**O**) Effect of FASstatin on PO-induced lipid accumulation (revealed by Nile Red staining) in TRIM56-depleted hepatocytes. si-SC, scrambled siRNA (*n* = 3). Scale bar: 50 μm. *P < 0.05, **P < 0.01, and ***P < 0.001 (**G**, **I**, and **J**).

Pharmacotherapies targeting NAFLD are an unmet medical need. Therapeutic targeting of protein degradation is an important strategy for NAFLD, given the nature of NAFLD as a spectrum of diseases characterized by nutrient surplus (18, 19). FASN is recognized as a master regulator of NAFLD pathogenesis, thus degradation of FASN protein expression or inhibition of FASN enzyme activity can lead to the suppression of hallmark events in NASH (e.g., fatty acid metabolism, fibrogenesis, and lobular inflammation) (20, 21). To identify lead compounds that inhibit lipid accumulation as a protype of FASN inhibitors (FASNi), we performed an AI-based screening of a drug library consisting of 1.2 billion small-molecule compounds (Figure 8A). After the screening, we selected 14 compounds for further validation using PO-challenged human hepatocytes (Figure 8B). FASNi-8 (referred to hereafter as FASstatin; chemical structure is shown in Figure 8C) stood out in this screening assay because of diminished lipid droplet formation in the human hepatocyte line. Molecular docking analysis suggested that FASstatin might have a binding mode with FASN protein (Figure 8D). SPR analysis revealed that FASstatin directly bound FASN (kDa = 1.6 × 10^–5^ M) (Figure 8E). As an alternative approach to validate the direct interaction between FASstatin and FASN, we generated FASstatin linked with a biotin tag (Bio-FASstatin). A pulldown assay indicated that FASstatin bound FASN (Figure 8F). However, only WT FASN could bind FASstatin, as mutation of 2 predicted key residues of FASN (Glu2251 and Tyr2343) yielded a negative binding mode with FASstatin (Supplemental Figure 3). We then explored the pharmacological actions of FASstatin. Our data demonstrated that FASstatin was not toxic to the human hepatocyte line at concentrations up to 40 μM (Figure 8G). We next compared the efficacy of FASstatin with the currently used FASN inhibitor C75 and observed that FASstatin had higher suppressive effects on FASN protein expression, comparable FASN enzyme inhibition, and higher efficacy in suppressing lipid droplet formation (Figure 8, H–K). Since TRIM56 is a E3 ligase that promotes FASN protein degradation, we sought to determine whether FASstatin promotes TRIM56-dependent FASN ubiquitination. Intriguingly, we observed that TRIM56-FASN interaction and TRIM56-mediated FASN K48-linked polyubiquitination were substantially increased by FASstatin treatment (Figure 8, L–N). Of functional relevance, FASstatin treatment reversed the PO-induced lipid accumulation triggered by *TRIM56* depletion (Figure 8O).

### FASstatin protects against NAFLD with good safety and oral bioavailability.

**Figure 9:**
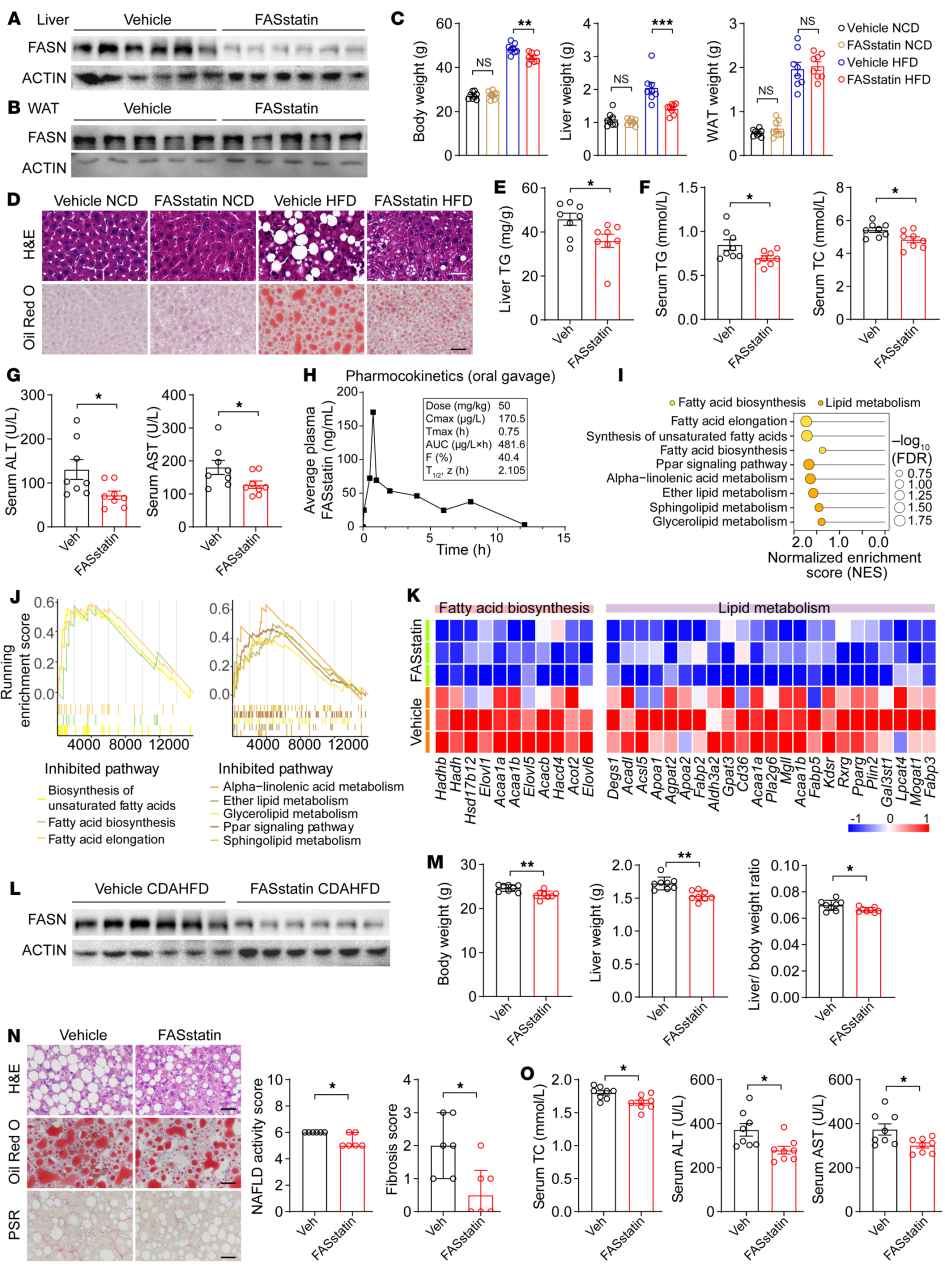
FASstatin protects against NAFLD and NASH with good safety and oral bioavailability. (**A** and **B**) The NAFLD model was established by feeding male C57BL/6J mice a HFD for 16 weeks. Thereafter, mice were orally administered vehicle or FASstatin (50 mg/kg/d) for an additional 8 weeks, concurrently with HFD feeding. FASN protein expression in livers and white adipose tissue (WAT) of NAFLD mice treated with vehicle or FASstatinin 2 groups of mice (*n* = 5–6, 6). (**B**) Western blot analysis of FASN protein expression in WAT of NAFLD mice treated with vehicle or FASstatin as described in A (*n* = 5). (**C**) Body weight, liver weight, and WAT weight of mice as described in A (*n* = 8). One-way ANOVA followed by Bonferroni’s post hoc test. (**D**) Representative images of H&E-stained (top) and Oil Red O–stained staining(bottom) of liver sections from 2 groups of miceNCD- or HFD-fed mice treated with vehicle or FASstatin for 8 weeks (*n* = 6). Scale bars: 50 μm. (**E**) TG content per gram of liver from the indicated groups of mice fed a HFDdetermination (*n* = 8). Two-tailed Student’s *t* test. (**F**) Serum levels of TG and TC in vehicle- and FASstatin-treated mice (*n* = 8). Two-tailed Student’s *t* test. (**G**) Serum levels of ALT and AST in vehicle- and FASstatin-treated mice (*n* = 8). Two-tailed Student’s *t* test. (**H**) Pharmacokinetic characterization of FASstatin in the plasma of C57BL/6J mice after a single dose of FASstatin administered via oral gavage (50 mg/kg). Plasma was harvested at the indicated time point, and the concentration-time curve (T_1/2_) was plotted (*n* = 3). The bioavailability factor (**F**) was calculated. (**I**) Pathway enrichment analysis of liver tissues from vehicle- or FASstatin-treated mice fed a HFD (*n* = 3). (**J**) GSEA analysis of the indicated pathways (*n* = 3). (**K**) Heatmap analysis of differentially expressed genes in liver tissues from HFD mice treated with vehicle or FASstatin (*n* = 3). (**L**) Effect of FASstatin on FASN protein expression in liver tissues from mice fed a CDAHFD (*n* = 6). Male C57BL/6J mice were fed a CDAHFD for 2 weeks before treatment with vehicle or FASstatin (50 mg/kg/d, i.g.) for an additional 4 weeks. (**M**) Effect of FASstatin on liver weight, body weight and the liver weight/body weight ratio in mice fed a CDAHFD as described in **L** (*n* = 8). Two-tailed Student’s *t* test. (**N**) Effect of FASstatin on liver pathology (H&E staining), hepatic steatosis (Oil Red O staining), and fibrogenesis (Picrosirius red staining of vehicle- and FASstatin-treated NASH mice). NAS and fibrosis score was calculated (*n* = 6). Scale bars: 50 μm. Mann-Whitney *U* test. (**O**) Effect of FASstatin on serum TC, ALT, and AST levels (*n* = 8). Two-tailed Student’s *t* test (**E**–**G**, **M**, and **O**). *P < 0.05, **P < 0.01, and ***P < 0.001 (**C**, **E**–**G**, and **M**–**O**).

To evaluate the therapeutic efficacy of FASstatin against NAFLD, NAFLD was established in mice fed a HFD for 16 weeks, and then the mice were orally administered FASstatin (50 mg/kg/d) while continuing the HFD for an additional 8 weeks. We selected the dose of FASstatin on the basis of a pilot study with escalating doses of FASstatin (0, 25, 50, 100 mg/kg/d) (Supplemental Figure 4). We found that liver and body weights increased after the HFD feeding. However, pharmaceutical intervention with FASstatin significantly decreased FASN protein expression in mouse livers but not in white adipose tissue (WAT) and reversed the HFD-induced increases in liver weight and body weights, but not WAT weight (Figure 9, A–C). Histologically, photomicrographs of H&E- and Oil Red O–stained sections revealed that micro- and macrovesicular fat deposition and hepatocellular ballooning were reduced in mice receiving FASstatin treatment (Figure 9D). Also, liver TG, serum TC, TG, ALT, and AST levels were lowered by FASstatin treatment (Figure 9, E–G). Next, we performed a pharmacokinetics study in the mice. Our data revealed that FASstatin was orally bioavailable and had a bioavailability of 40.4% when given orally at a dosage of 50 mg/kg (Figure 9H). We then assessed and characterized the safety profile of FASstatin in mice. Our data suggest that FASstatin did not affect the levels of urea or creatine kinase–MB (CK-MB), established biomarkers associated with kidney and myocardial injury. No apparent histological difference was observed between vehicle- and FASstatin-treated mice (repeated dosing of FASstatin at 50 mg/kg/d for 8 weeks) (Supplemental Figure 5, A and B). In the acute toxicity test, mice could tolerate FASstatin at 1.75 g/kg after single-dose administration. FASstatin (single dose at 1.75 g/kg) did not affect body weight or the weights of liver, kidney, heart, lung, or spleen. FASstatin also had no significant effect on the levels of ALT, AST, Crea, CK, or lactate dehydrogenase (LDH), established biomarkers associated with liver, kidney, and myocardial injury. We observed no apparent histological difference or tissue damage between the vehicle- and FASstatin-treated mice (single dose at 1.75 g/kg) (Supplemental Figure 5, C–E). To further investigate the molecular mechanisms underlying the histopathological improvements mediated by FASstatin, we performed transcriptomic profiling of vehicle- and FASstatin-treated liver tissues. Our results indicate that enriched pathways in lipid metabolism and fatty acid biosynthesis were attenuated by FASstatin treatment (Figure 9, I–K).

To further explore the therapeutic potential of FASstatin in the advanced stage of NAFLD, we established a NASH model by feeding mice a choline-deficient HFD (CDAHFD), a well-established model that exhibits characteristics of human NASH including hepatic steatosis, liver damage, and fibrosis (22, 23). Our data demonstrate that FASstatin decreased FASN protein expression (Figure 9L) and significantly reduced body and liver weights (Figure 9M), hepatic steatosis, fibrosis, and inflammation, and serum ALT and AST activity (Figure 9, N and O). Based on the weight of evidence presented above and its safety and efficacy profile, FASstatin may be effective as an orally bioavailable and well-tolerated lead compound to block NASH development.

## Discussion

NAFLD is the most prevalent form and etiological factor of metabolic liver disease, affecting 38% of the general population (24). The number of people affected is ever increasing due to the silent nature of this disease and insufficient attention to its prevalence. The associated health care burden of NAFLD continues to rise worldwide including in China (25, 26). However, there are still no effective pharmacotherapies approved by the FDA to treat NAFLD despite the fact that weight loss and lifestyle modification are beneficial (20, 21). Although some drug candidates have shown promising therapeutic effects in preclinical studies and have entered the therapeutic pipeline, most of these drugs have failed to achieve histological endpoints of NASH (20, 21). Given the high prevalence and silent nature of NAFLD as well as adverse clinical outcomes necessitating liver transplantation, there is an unmet medical need to identify effective therapeutic targets and pharmacotherapies for NASH. Regulatory pathways of protein homeostasis represent a promising avenue to address the metabolic-overload nature of NAFLD. FASN is a multifunctional target in NAFLD because of its purported role in DNL, liver inflammation, and fibrogenesis. Targeted degradation of FASN by E3-ligase is an appealing therapeutic approach in NAFLD therapeutics. Here, we describe a TRIM56/FASN axis that regulates hepatocyte lipid accumulation and lipotoxicity. Our data suggest that TRIM56 downregulation may thus serve as a converging point by which overnutrition and steatosis contribute to liver damage. In addition, AI-assisted drug discovery yielded the identification of a small-molecule inhibitor of FASN, FASstatin, which boosted TRIM56-FASN interaction and increased TRIM56-driven FASN degradation and thus ameliorated NAFLD pathologies in mice with good efficacy, safety, and pharmacokinetics profiles. These results suggest the potential utility of therapeutically targeting the TRIM56/FASN axis in NAFLD.

Using multilayer screening, we identified the conserved enrichment of the TRIM family in mouse and human NAFLD. TRIM is a protein family belonging to E3 ubiquitin ligases. TRIM family proteins can be induced by virus infection and are recognized as important regulators of antiviral defense and host innate immunity (27). Hepatocytes are essential for the innate immune response by sensing and responding to physiological, pathophysiological, and environmental cues (28). Emerging evidence has convincingly demonstrated that hepatocytes are immune sentinels that sense and respond to immune, inflammatory, and metabolic insults. In the scenario of metabolic liver disease, it has been reported that loss-of-function mutations in the peroxisomal/nuclear protein TRIM37 cause a monogenic multiorgan disorder characterized by metabolic syndrome (29, 30). FASN is one of the most attractive targets for NAFLD, given its role in controlling key mechanisms of NASH, i.e., hepatic DNL, inflammatory, and fibrogenic pathways (31, 32). However, the regulatory mechanisms of FASN in NAFLD and potential therapeutic strategies targeting FASN remain largely unknown. Sorting nexin 8 (SNX8) was found to mediate FASN protein degradation by recruiting TRIM28 and enhancing TRIM28-FASN interaction (33). In addition, Yan et al. reported that TRIM8 expression is increased in liver tissues from patients with NAFLD/NASH. Mechanistic studies have revealed that TRIM8 promotes NAFLD/NASH development by direct interaction with and ubiquitination of TGF-β–activated kinase 1 (TAK1), thus promoting TAK1 phosphorylation and the ensuing activation of the JNK/p38 and NF-κB signaling pathways (34). Likewise, a more recent study has shown that TRIM16 attenuates hepatocyte steatosis and inflammation in a mouse NASH model by directly interacting with the phosphorylated form of TAK1 and promoting its degradation, leading to suppressed NASH development (16). Two more recent studies have demonstrated that TRIM31 alleviates NAFLD and NASH pathologies by targeted degradation of rhomboid 5 homolog 2 (Rhbdf2) (35) and TAK1 (36) in the liver. TRIM31 is also responsible for the antifibrotic effects of mulberrin (a bioactive phytochemical from the traditional Chinese medicine Ramulus Mori) in CCl_4_-induced liver fibrosis (37). These lines of evidence illustrate the therapeutic potential of targeting TRIM family members in different stages of NAFLD. By using unsupervised siRNA library screening of the TRIM family proteins involved in hepatic steatosis, we found that TRIM56, an established RING-type E3 ubiquitin ligase in innate antiviral immunity (15, 27), was the most notable suppressor of PO-induced lipid accumulation.

Using unbiased protein interactomics screening and molecular validation, we found that TRIM56 directly interacted with FASN, a key lipogenesis factor driving hepatic steatosis in NAFLD/NASH. The increased lipid accumulation in *Trim56*-KO hepatocytes promoted by PO treatment could be prevented by genetic depletion or pharmacological inhibition of FASN, suggesting that FASN is a key downstream factor that regulates *Trim56* deficiency in hepatocytes. Overall, the spectrum of research data gleaned for TRIM56 was directionally congruent with the hepatoprotective role of TRIM56 in counteracting the pathophysiology of NAFLD.

Recently, some FASN inhibitors, such as platensimycin (38), have been shown to intervene in NAFLD progression in mouse models. Fortunately, some FASN inhibitors, such as TVB-2640 (32), a first-in-class and orally bioavailable FASN inhibitor, has undergone phase II clinical trials that have shown significant effects in reducing liver fat and improving biochemical, inflammatory, and fibrotic biomarkers after 12 weeks of treatment. FT-4101 (39), another FASN inhibitor, is also in clinical development. These promising findings gleaned from preclinical and clinical studies prompted us to identify potential FASN inhibitors. To effect clinical translation, by using a structure-guided approach, we discovered a small-molecule inhibitor of FASN, FASstatin. It outperforms C75, the first-generation FASN-targeting drug, in promoting FASN degradation. The effect of FASstatin at the tested concentration possibly derives from dual mechanisms including inhibition of FASN enzyme activity as well as FASN protein degradation. Based on the pharmacology data for FASstatin, this drug candidate appears more likely to be a proteolysis-targeting drug to reduce FASN protein expression. Further in-depth studies are needed to validate the contribution of FASN enzyme inhibition versus protein degradation to the observed pharmacological actions of FASstatin in vivo. FASstatin boosts the interaction between TRIM56 and FASN, leading to FASN inhibition in hepatocytes. Furthermore, our in vivo data demonstrate that inhibition of FASN by FASstatin reduced both circulating and hepatic levels of TC and TGs. Most important, FASstatin was orally bioavailable and well tolerated in mice. Oral administration of FASstatin in HFD-induced NAFLD in mice led to improved NAFLD pathologies. These pharmacological features suggest that the effects of FASstatin on NAFLD could be different from those of acetyl-CoA carboxylase (ACC) inhibitors, which have been shown to lower hepatic TG levels but to increase circulating TG levels (40, 41).

In light of the multifaceted roles of FASN in fatty acid synthesis, lipid metabolism, and infection, pharmacological inhibition of FASN could also offer other extended protective effects, such as antiaging (42), inhibition of HCC (43), as well as SARS-CoV-2 replication (33), etc. Additional work will be necessary to verify whether FASstatin could also exert pleiotropic protective effects in these diseases. Beyond its canonical role in innate immunity (such as the cGAS/STING pathway) (44, 45), TRIM56 may also execute pivotal metabolic regulatory effects in an immunity-independent manner. In addition, only male mice were used in the current study. However, since female mice, typically resistant to HFD-induced obesity and NAFLD, can develop full NAFLD characteristics under thermoneutral housing conditions (46), further studies are warranted to elucidate the effects of FASstatin on NAFLD in female mice. Last, the doses of FASstatin in cultured cells were much higher than the drug concentration in mouse serum. This is because in vivo and in vitro systems are different, and serum drug concentrations cannot be directly equivalent to the drug concentrations in cultured cells. The duration and frequency of administering the drug, its metabolites, solubility, permeability, the cell type involved, and the liver microenvironment are all important factors affecting the efficacy of the drug. Detailed characterization of the FASstatin pharmacology and tissue distribution is warranted in future studies.

In summary, we demonstrate that hepatocyte-derived TRIM56 played a central role in maintaining metabolic homeostasis in the context of NAFLD. Thus, our work provides the proof of concept that pharmaceutical intervention of the TRIM56/FASN axis could be a clinically translatable approach to rescue the systemically deregulated lipid metabolism observed in NAFLD.

## Methods

Additional details on methods can be found in the Supplemental Methods. 

### Animal study.

Mice were randomized into each group by randomization table.

### Collection of human tissue samples.

Human fatty liver samples were obtained from individuals with NAFLD who underwent a liver biopsy. Patients with NAFLD due to viral infection, autoimmune hepatitis, or excessive alcohol use (140 g/week for men or 70 g/week for women) were excluded for analysis. The NAFLD-free control samples were obtained from normal donors undergoing liver surgery. All of the individuals enrolled in this study were free of drug abuse, hepatitis virus infection, and excessive alcohol consumption. Liver histology was evaluated by 2 independent liver pathologists blinded to the patients’ demographic and other clinical data. The NAFLD activity score (NAS) was evaluated according to the NASH Clinical Research Network Scoring System criteria (47, 48).

### Statistics.

Data are presented as the mean ± SEM if data were normally distributed. For data that were not normally distributed, data are presented as the median and IQR. Before statistical analysis, data normality was assessed by the Shapiro-Wilk test. For data that were normally distributed, an unpaired 2-tailed Student’s *t* test was used to calculate statistical differences between 2 groups. For comparisons among multiple groups, 1-way ANOVA followed by Bonferroni’s post hoc test was used for data with homogeneity of variance. For data that did not follow normal distribution, a nonparametric test (Mann-Whitney *U* test for 2 groups; Kruskal-Wallis test for multiple groups) was applied for statistical analysis. In the 1-way ANOVA, Tamhane’s T2 analysis was used when heteroscedasticity was observed. Statistical analysis was performed using SPSS Statistics version 21.0. A *P* value of less than 0.05 was considered statistically significant. All images shown without biological replicates are representative of a minimum of 3 independent experiments.

### Study approval.

Human sample collection and use complied with the ethics committees of Renmin Hospital of Wuhan University (approval no. WDRY2019-K011) and the First Affiliated Hospital of University of Science and Technology of China (approval no. 2023KY-383). The study protocol conforms to the ethics guidelines of the 1975 Declaration of Helsinki. Written informed consent was obtained from each participant for the collection of samples. All animal studies were performed in accordance with the IACUC of the University of Science and Technology of China (approval no. USTCACUC212401038) and the Renmin Hospital of Wuhan University (approval no. WDRM20201207A). Reporting of the animal studies was done in compliance with Animal Research: Reporting of In Vivo Experiments (ARRIVE) guidelines 2.0 listed in the Enhancing the QUAlity and Transparency Of health Research (EQUATOR) Network library.

### Data availability.

All RNA-Seq data supporting the findings of this study have been deposited in the NCBI’s Sequence Read Archive (SRA) under accession numbers PRJNA1011487, PRJNA1011905, and PRJNA1011918. All remaining data that support the findings of this study are available in the main text or the supplemental materials. See the Supplemental Supporting Data Values file for values underlying the data presented in each graph and as means in the figures.

## Author contributions

JW conceptualized the study. SX, XW, SW, and MX designed the study methodology. SX, XW, SW, MX, MC, JF, JG, TT, XC, JZ, Zhihua Wang, Zhenya Wang, and YY performed experiments. JW, YXJ, and SX acquired funding. SX, XW, SW, XM, HY, ST, XM, TF, Zhihua Wang, and Zhenya Wang were responsible for project administration. JW, SX, and XJZ supervised the study. SX wrote the original draft of the manuscript. WX, FX, JY, SL, and XJZ reviewed and edited the manuscript.

## Supplementary Material

Supplemental data

Unedited blot and gel images

Supporting data values

## Version 1 01/11/2024

In-Press Preview

## Version 2 03/01/2024

Electronic publication
