# Supplementary material for: TRIM56 protects against nonalcoholic fatty liver disease by promoting the degradation of fatty acid synthase
Source: J Clin Invest. 2024 Jan 11;134(5):e166149. doi: 10.1172/JCI166149 (PMC10904058; doi:10.1172/JCI166149)
Supplement: Supplemental data [file jci-134-166149-s276.pdf]

Supplemental Materials for

TRIM56 protects against non-alcoholic fatty liver disease via promoting the degradation of fatty acid synthase

Xu *et al*

*Correspondent author: Jianping Weng, wengjp@ustc.edu.cn

The PDF file includes:

Supplemental Materials and Methods
Figure S1 to S5
Tables S1 to S2
Scheme 1

Supplemental Materials and Methods

Mice, diet, husbandry and interventional strategies

Male C57BL/6J mice (6-8 weeks of age) were purchased from GemPharmatech Co., Ltd (Nanjing, China) or Vital River (Beijing, China). In this study, only male mice were used because NAFLD is a sex dimorphic disease and estrogen has been shown to be a key factor in NAFLD [1]. After one week of acclimatization, all mice were housed in isolated ventilated cages (IVC) in a temperature-and humidity-controlled animal facility with a 12/12 h light/dark cycle at 22-26ºC. Mice had free access to food and water *ad libitum*.

Male mice were fed with normal chow diet (NCD) or a high-fat diet (HFD, 60 kcal% fat, 20 kcal% protein, 20 kcal% carbohydrate; D12492; Research Diets, New Brunswick, USA) for 16 or 24 weeks to establish NAFLD as indicated in the figure legends. When experimental endpoint was achieved, blood was withdrawn retro-orbitally and serum was collected for analysis of blood biochemistry parameter. Liver tissues were harvested, snap-frozen in liquid nitrogen, and processed for isolation of RNA or total protein. A fraction of liver tissues embed in OCT compound were processed for staining of lipid droplet by using Oil Red O and H&E staining.

In the acute toxicity experiment, male C57BL/6J mice were treated with a single dose of FASstatin (1.75 g/kg/d, i.g.) according to U.S. FDA guidelines. Mouse behavior and phenotypic observations were recorded. 14 days after dosing with FASstatin, tissue weights were measured and histology was processed.

To assess the therapeutic role of FASstatin in established NAFLD, a mouse NAFLD model was established by feeding male C57BL/6J mice (6-8 weeks) with a HFD (D12492; Research Diets) for 16 weeks [2]. Thereafter, mice were orally administered with vehicle (0.5% methyl cellulose) or FASstatin (50 mg/kg/d, in vehicle) for an additional 8 weeks concurrently with HFD feeding. The chemical synthesis of FASstatin is described in Scheme 1. To assess the safety profile of FASstatin in mice, male C57BL/6J mice were orally administered with vehicle or FASstatin (50 mg/kg/d) for 16 weeks. To further explore the therapeutic potential of FASstatin in advanced stage of NAFLD-NASH, we established NASH model by feeding mice with a choline-deficient HFD (CDAHFD), a well-established model which exhibits characteristics of human NASH including hepatic steatosis, liver damage and fibrosis [2,3]. Male C57BL/6J mice (6weeks) were fed with CDAHFD (A06071302; Research Diets, New Brunswick, USA) for 2 weeks before treatment with FASstatin (50 mg/kg/d) for an additional 4 weeks. The dosage of FASstatin was determined by pilot studies. In the pilot study, we feed male C57BL/6J mice for CDAHFD for 2 weeks before treatment with vehicle (0.5% methyl cellulose) or three escalating doses of FASstatin (25, 50, 100 mg/kg/d, *p.o.*) for additional 4 weeks concurrent with feeding with CDAHFD. 24 h after the last dose, liver samples were collected. The sample size for each experimental group was pre-determined with an α value of 0.05 and a 1-β value of 0.8.

All the animal experiments were approved by the Animal Care and Use Committee of the University of Science and Technology of China (USTC) and the Institutional Animal Care and Use Committee of Renmin Hospital of Wuhan University. Animal handling procedures were humane and the study protocol conform to the Animal

Research: Reporting of In Vivo Experiments (ARRIVE) guidelines as well as the Guide for the Care and Use of Laboratory Animals stipulated by NIH, USA.

Generation of genetically modified mice

Trim56-knockout (*Trim56*-KO) mice were generated using a CRISPR/Cas9 technology in the C57BL/6J background. A single guide RNA (sgRNA, sequence: GTGCCTGTACCCGCCGAAGGGG TGG) targeting exon3 of *Trim56* were designed by an online CRISPR design tool (http://chopchop.cbu. uib.no/). The target site sequence was inserted into pUC57- sgRNA vector (51132, Addgene). Purified the sgRNA and Cas9 mRNA which in vitro transcription from Cas9 expression plasmid (44758, Addgene) were mixed and injected into the fertilized eggs of C57BL/6J mice. Founder with indels in the target gene was amplified by PCR analysis with the following primers: F1: 5'- TGAGCAGCGATTTCCTAGCC-3'; R1: 5'-GCATAAGTCGTCGGCACAGT-3'. Founder mice were mated with C57BL/6J female mice, and their positive offspring were crossed to obtain *Trim56*-KO mice. Genotyping of *Trim56*-KO mice were performed with PCR analysis using the primer pairs (forward: TGAGCAGCGATTTCCTAGCC; reverse: GCATAAGTCGTCGGCACAGT). All products were confirmed by sequencing. Founder mice were mated with C57BL/6J female mice and several rounds of crossbreeding was performed to obtain homozygous *Trim56*-KO mice. To generate *Trim56* liver-specific knockout mice, 5×10^{11} vg AAV8 carrying three sgRNA sequences of mouse *Trim56* gene (AAV8-U6-sgRNA1-U6-sgRNA2-U6-sgRNA3-m *Trim56*-TBG-Cre, Weizhen Bio; Jinan, China) was injected into C57BL/6J-Gt(ROSA26)Sortm1(CAG-LNL-Cas9)smoc mice (NM-KI-00038, Shanghai Model Organisms Center, Shanghai, China) via tail vein. The efficiency of gene knockout was evaluated by Western blot. Hepatic overexpression of *Trim56* (*Trim56*-HepOE) in mice were achieved using the Sleeping Beauty transposon system as described previously [4], with slight modifications from published literature [5, 6]. In brief, male 8-week-old C57BL/6J mice were treated with a DNA solution (in PBS) containing 2 µg of SB100 transposase-encoding plasmid (pCMV-SB100) concurrent with 50 µg of pT3-alb- 3xFlag-m*Trim56* via hydrodynamic tail vein injection. Mice in the control group were injected with the pT3-alb-3×Flag-control plasmid.

Cell isolation and culture

Primary hepatocytes were isolated from 8- to 10-week-old male C57BL/6J mice or *Trim56*-knockout mice according to published literature [4]. In brief, mice were anaesthetized with 1% sodium pentobarbital. Then, mouse liver was perfused with perfusion buffer (137 mM NaCl, 5.4 mM KCl, 0.5 mM $NaH2PO4$, 0.4 mM $Na2HPO4$, 4.2 mM $NaHCO3$, 0.5 mM EGTA, 5 mM Glucose, pH 7.4) (5 ml/min) followed by perfusion with Liver Digestion Medium containing collagenase (17701-034; Thermo Fisher Scientific; Waltham, MA) (2.5 ml/min) via the portal vein. The liver was then transferred to rat tail collagen pre-coated cell culture dishes, and cells were filtered through 70 µm Cell Strainers (BD Falcon) to remove undigested debris. The filtrate was washed twice with ice-cold DMEM and washed cell pellet was centrifuged at 200 g for 3 min. Isolated hepatocytes were plated on rat tail collagen pre-coated cell culture

dishes and cultured with DMEM supplemented with 10% FBS and 1% penicillin-streptomycin antibiotics (15140-122; Thermo Fisher Scientific, Waltham, MA) at 37 °C in a humidified incubator with 5% CO_2.

Human hepatocyte Huh7 cells (human hepatic carcinoma cell line), HepG2 cells (human hepatic carcinoma cell line), human embryonic kidney (HEK) 293T cell lines were purchased from the National Collection of Authenticated Cell Cultures (Shanghai, China). Cells were cultured in a 5% CO_2 incubator with controlled humidity. All cells were cultured in DMEM supplemented with 10% FBS and 1% penicillin-streptomycin unless specified otherwise. All the cell lines were validated and exempt from mycoplasma, bacterial and fungi contamination. To mimic hepatic steatosis/inflammation/lipotoxicity in cultured cells, or mouse primary hepatocytes were stimulated with palmitic acid (PA; 0.5 mM; P0500; Sigma-Aldrich; St. Louis, MO) and oleic acid (OA; 1.0 mM; O-1008; Sigma-Aldrich; St. Louis, MO) (dissolved in 0.5% fatty acid–free BSA) for 18 h using fatty acid-free BSA (0.5%; BAH66-0100; Equitech Bio; Kerrville, TX) as vehicle control. For FASN inhibitory experiment, hepatocytes were treated with FASN inhibitor C75 (20 μM; TargetMol, Shanghai, China) or shFasn to specifically inhibit FASN activity or expression. Experiments in cultured cells were performed at least three times independently unless specified otherwise. All cell lines were verified to be free of contamination with mycoplasma, virus or fungi.

Pharmacokinetic studies

Eight-week-old male C57BL/6J mice were administered with a single dose of FASstatin (1 mg/kg for intravenous injection; 50 mg/kg for oral gavage), and blood samples were collected, then plasma of mice were separated after the indicated time points. FASstatin concentration in mouse plasma was extracted with chloroform/methanol (8:2), and the lower organic layer were dried under nitrogen, then dissolved in methanol. Liquid chromatography-mass spectrometry/mass spectrometry (LC-MS/MS) analysis was performed on the AB SCIEX Triple Quad 4500 triple quadrupole mass spectrometer with SRM as described previously [7]. Analyst 1.6.1 was used for the quantitative analysis. The pharmacokinetic parameters were calculated through non-compartmental pharmacokinetic analysis using Phoenix WinNonlin software (version 6.3, Pharsight) with the linear/log trapezoidal rule [7].

Determination of hepatic level of TC and TG

At the end of experiment, mice were sacrificed for analysis. Liver tissues were collected, homogenized and centrifuged. Cleared supernatant were used for analysis of hepatic triglyceride (TG) content using commercially available kits (290-63701 for TG; Wako; Tokyo, Japan). Final level of TG was normalized with liver tissue weight.

Blood biochemistry

After blood was withdrawn retro-orbitally, serum was prepared for analysis of TG, TC, alanine aminotransferase (ALT) and aspartate aminotransferase (AST) using an automatic ADVIA 2400 Biochemistry Analyzer (Siemens, Tarrytown, NY). To probe the effect of FASstatin on the biochemical parameters associated with tissue injury, the

levels of ALT (marker of liver injury), AST (marker of liver injury), CK- MB isoform (marker of myocardial injury), and UREA (marker of kidney injury) was determined using an automatic ADVIA 2400 Biochemistry Analyzer (Siemens, Tarrytown, NY).

Histological analysis

After sacrifice, mouse liver tissues were fixed by 4% PFA overnight at 4 °C embedded for paraffin section (sectioned at 8 μm by Leica RM2235) and H&E staining using commercially available kits (6765001, 6766010, Thermo Scientific). Another fraction of liver tissue was embedded in OCT compound and cryosections were prepared for Oil Red O staining (O0625; Sigma-Aldrich; St. Louis, MO). For collagen staining (index of extent of liver fibrosis), paraffin sections of liver were stained with PicroSirius Red (PSR; (ab150681, Abcam) staining, and the histological images were acquired with a light microscope (Olympus; Tokyo, Japan). At least 6-8 different optical fields were taken for individual animal. At least 6-8 different optical fields were taken for individual animal. To evaluate the safety profile of FASstatin in normal control mice, major organs/tissues were collected for histological H&E staining.

siRNA library screening of TRIM family members

The siRNA library of 73 TRIM family members was custom purchased from Ribo Bioscience Inc (Guangzhou, China). Silencing efficiency of siRNAs in the library was verified by real-time PCR analysis. siRNA at 50 nM was transfected in human hepatocyte cell line for 48 h in the presence of PO treatment. At experimental endpoint, Nile Red-based fluorescent staining was performed and mean fluorescence intensity of individual treatment was calculated by normalization with cell number.

Confocal microscopy

Liver sections were permeabilized with 0.1% Triton X-100 in PBS. After rinsing twice with PBS, sections were blocked with normal goat serum for 30 min. Then, primary antibodies were incubated with section overnight at 4 °C before incubation with a fluorophore-conjugated secondary antibodies (Alex Flour-546 goat anti-rabbit IgG (H+L); A11035; Invitrogen; Carlsbad, CA) for 1 h at room temperature in the darkness. After that, sections were mounted with Prolong Gold-Anti-Fade mounting media from Invitrogen (P36930, Carlsbad, CA). A fluorescence microscope (Olympus; Tokyo, Japan) or Operetta CLS high-content analysis system (PerkinElmer, Waltham, MA) was used to acquire immunofluorescent images. For confocal microscopy in cultured cells, human or mouse hepatocytes at sub-confluency were transfected with the indicated plasmids or siRNAs or adenoviruses or treated with indicated compounds for 24 h. Cells were permeabilized with 0.1% Triton X-100 in PBS and blocked as described above. Cells were then incubated with primary and secondary antibodies using DAPI as counterstaining dye. Images were acquired with a confocal laser scanning microscope (TCS SP8; Leica; Wetzler, Germany) or Operetta CLS high-content analysis system (PerkinElmer, Waltham, MA).

Nile Red and BODIPY staining

For Nile Red staining, HepG2, Huh7 cells and mouse primary hepatocytes were treated with PO for 18 h. Then, the cells were fixed with 4% paraformaldehyde (PFA) and stained with Nile Red (N1142, Invitrogen, Carlsbad, CA) or BODIPY (D3922, Invitrogen, Carlsbad, CA). Intracellular lipid accumulation was visualized and quantified by a laser scanning confocal microscope (TCS SP8; Leica; Wetzler, Germany) or an Operetta CLS high-content analysis system (PerkinElmer, Waltham, MA).

Artificial Intelligence (AI)-assisted screening of FASN inhibitors

The FASN thioesterase active domain (2200-2511aa, pdb:2px6) is molecularly docked (Autodock Vina 1.1.2 [8]) with 1.2 billion structurally novel compounds in the synthesizable compound library to generate about 100,000 virtual drugs. Before docking, PDB file (2px6 chain R, available from: http://www.rcsb.org/) were converted to the PDBQT format as macromolecules before virtual screening. All heterogeneous atoms and the FASN ligand were removed and 2px6 chain R was selected for subsequent virtual screening for 1.2 billion compounds. Then, the "triple integral collaborative AI-filtering scoring system" was used to obtain candidate compounds with higher scores, followed by prediction of druggability of candidate compounds by the "Big data-AI-ADMET prediction system". Finally, positive hit compounds were filtered out with higher druggability ranking score. Protein–ligand interactions were visualized using Pymol version 1.7.4.5. The amino acid residues of FASN protein close to the hit ligands (≤1 Å) were highlighted as potential interactive residues involved in the protein–ligand interaction.

Quantitative PCR analysis

Total RNA was extracted with ESscience RNA Quick-Purification Kit (Yishan Biotech, Shanghai, China) followed by cDNA synthesis using PrimeScript RT Reagent Kit from Vazyme (R212-01, Nanjing, China). Quantitative real-time PCR assays were performed with AceQ SYBR Green PCR Master Mix (Q121-02, Vazyme; Nanjing, China) in a LightCycler 480 Cycling System (Roche, Basel, Switzerland) per the manufacturer's instructions. Housekeeping genes beta-actin, or GAPDH was used for normalization. Fold change relative to respective controls was calculated using the $2^{-\Delta\Delta Ct}$ method. The primers are designed using PrimerQuest Software and synthesized in Sangon Biotech (Shanghai, China). Sequence of primers used are listed in Supplemental Table 1.

Western blot analysis

Whole cell lysates from tissues and cultured cells were collected by lysis with RIPA lysis buffer (P0013E; Beyotime, Shanghai, China) before sonication on ice. Cell lysate was cleared by centrifugation at 4 degree at 12,000g for 30 min. Total protein concentration was quantified using a BCA kit from Thermo Fisher Scientific (23225, Waltham, MA) by absorbance reading and concentration calculation using BSA as the standards. Then, same amounts of total proteins were separated by 8-10% SDS-PAGE (depending on the molecular weight of indicated protein) and transferred to PVDF

membranes (IPVH00010; Millipore, Billerica, MA) or nitrocellulose membrane (10600002, Amersham). The membranes were then blocked with 5% skimmed milk in 1×TBST before incubation with primary antibodies overnight at 4 C, followed by incubation with HRP-conjugated secondary antibodies. Protein signal was then detected using an ECL kit (170-5061; Bio-Rad; Hercules, CA) and visualized in a ChemiDoc MP Imaging System (Bio-Rad, Hercules, CA). The primary and secondary antibodies used and vendor information are listed in Supplemental Table 2.

Recombinant proteins

The human-derived FASN and TRIM56 recombinant plasmids were synthesized in GENEWIZ Co., Ltd. (Suzhou, China). The thioesterase domain of FASN protein, 2200-2510aa, containing 933 bases and the full-length TRIM56, 1-755aa, containing 2265 bases, were both recombinantly cloned into the pET28a vector with a 6×His-tag at the carboxyl terminus. Codon optimization was performed using the GenSmart software to facilitate better protein expression in Escherichia coli. The procedures for protein expression and purification were reference to previous research methods. In short, the recombinant plasmids were transformed into BL21 (DE3) strains, and *Escherichia coli* was induced at 16°C for 24 hours with 400 μmol of Isopropyl-β-D-thiogalactopyranoside (IPTG). After 200W ultrasound for 30 min to lyse *Escherichia coli*, protein purification was performed using a NiNTA column, and the protein was ultimately preserved in a buffer containing 20 mmol/L Tris-HCl (pH 8.0), 200 mmol/L NaCl, and 5% glycerol, and stored at -80°C for later use.

Surface plasmon resonance for affinity determination

The SPR study method was described in our previous report[9, 10]. Briefly, in this experiment, the Biacore T200 (GE Healthcare) was used to measure the KD values of protein–protein and protein–ligand interactions. The CM5 chip (GE Healthcare) was used to couple the target protein. Before coupling the protein, the chip was first activated with 1-ethyl-3-(3-dimethylaminopropyl) carbodiimide (EDC, GE Healthcare) and N-hydroxysuccinimide (NHS, GE Healthcare) for 60 seconds at a flow rate of 10 μL/min. Then, the FASN protein was diluted to approximately 50 mg/mL with 10 mmol/L sodium acetate buffer (pH 4.5) and injected into the chip at a flow rate of 10 μL/min for 120 seconds to enrich the channel. The coupling amount of FASN protein in a single channel was approximately 10000 RU. Finally, the channel was blocked with ethanolamine at a flow rate of 10 μL/min for 120 seconds. For the affinity measurement of FASN and TRIM56, we diluted the TRIM56 protein from 20 μg/mL to 0.0390625 μg/mL by using the double dilution method and flowed the protein from low to high concentrations through the chip channel with or without FASN protein at a flow rate of 30 μL/min for 180 seconds. After each concentration point was flowed through, the chip was regenerated with NaOH at a flow rate of 30 μL/min for 60 seconds, and then real-time recording and data storage were performed. At the same time, molecular weight adjustment and solvent correction were used to eliminate nonspecific binding and molecular effects. For the affinity measurement of FASN and compounds, we diluted the compound from 20 μM to 0.0195 nM in 11 concentrations and flowed the

small molecule from low to high concentrations through a chip containing the target protein channel or not at a flow rate of 30 μL/min for 180 seconds. After each concentration point was flowed through, the chip was regenerated with NaOH at a flow rate of 30 μL/min for 60 seconds, and then real-time recording and data storage were performed. Finally, all data were analysed and sorted in Biacore T200 analysis software (GE Healthcare).

Immunoprecipitation assays

Immunoprecipitation assays were performed as we described previously [11]. Briefly, HEK293T and human hepatocyte cell lines with indicated treatments were lysed with IP lysis buffer (20 mM Tris-HCl, pH 7.4; 150 mM NaCl; 1 mM EDTA; and 1% NP-40) containing protease inhibitor cocktail from Sigma (P8340, St. Louis, MO). After 4 ºC centrifugation at 12,000 g for 10 min, cleared supernatants were incubated with protein A/G agarose beads (Millipore) and the indicated anti-tag antibodies or primary antibodies overnight at 4 ºC. For endogenous immunoprecipitation assays, cells were incubated with the indicated primary antibody targeting endogenous proteins. The beads were then washed with IP lysis buffer for 3-4 times. After last wash, interacting proteins were eluted with 1×SDS loading buffer by boiling at 95 ºC for 10 min. Finally, western blotting was performed as mentioned before.

Ubiquitination assay

Cells were transfected with specific plasmids at 80% density followed by drug exposure or indicated treatments. 10 μM MG132 was added to avoid proteasome-mediated protein degradation for 6 h before harvest. Cells were washed with ice-cold PBS, then whole cell lysate was collected and clarified by centrifugation. Cleared lysates was collected for IP using indicated antibodies coupled with protein A/G beads. Bound proteins were eluted with 1×SDS loading buffer, boiled and subject to western blot using indicated antibodies.

FASN enzyme activity assay

FASN is a key enzyme in fatty acid synthesis, catalyzing acetyl-coA and malonyl-coA to produce long-chain fatty acids. FASN catalyzes the production of long-chain fatty acids and $NADP^+$ from acetyl CoA, malonyl CoA and NADPH. NADPH has a characteristic absorption peak at 340 nm, while NADP+ does not. After treatment with vehicle (0.1%DMSO) or FASstatin (20 μM) or C75 (20 μM) for 6 hours, the huh7 cell is harvested. The enzyme activity of FASN was calculated by detecting the rate of decrease in absorption at 340 nm with CheKine™ Micro Fatty Acid Synthetase (FAS) Activity Assay Kit (Abbkine #KTB2240). 1 nmol NADPH oxidated per min in 1 mg tissue protein reaction system is defined as a unit of enzyme activity

Screening of candidate protein family in mouse and human NAFLD samples

The gene set of protein families were obtained from UniProt database and the data of Protein-Protein Interaction (PPI) was download from STRING database. For each protein family, interacting proteins with score >= 400 were used to construct the gene

set of interacting proteins of this protein family. The expression trend of constructed gene sets in NAFLD databases was explored by gene set enrichment analysis (GSEA). Gene sets with FDR values < 0.25 were considered statistically significant. The intersection of gene sets enriched in NAFLD group in all human NAFLD databases and gene sets enriched in HFD group in all mouse NAFLD databases was considered as candidate protein families.

RNA-sequencing

Total RNA from cultured cells and liver tissues was extracted using TRIzol Reagents (Thermo Scientific). RNA concentration and quality was assessed with Nanodrop. RNA was further processed for constructing cDNA libraries. Single-end cDNA libraries was constructed using MGIEasy RNA Library Prep Kit (1000006384; MGI, Shenzhen, China) and sequenced on a BGISEQ 500 instrument (MGI Tech Co., Ltd) with a read length of 50 bp. The reads were mapped to Ensembl mouse (mm10/GRCm38) reference genomes by HISAT2 software. StringTie was used to calculated Fragments Per Kilobase of exon model per Million mapped fragments (FPKM) value. DESeq2 (version 1.2.10) was used for differential gene expression analysis.

Gene set enrichment analysis (GSEA)

Each KEGG pathway term and involved genes were defined as gene sets, and GSEA was implemented on the Java GSEA (version 3.0) platform with the 'Signal2Noise' metric to generate a ranked list and a 'gene set' permutation type. Gene sets with FDR values < 0.25 were considered statistically significant. The expression levels of leading genes in the enriched pathways of GSEA was exhibited by heatmap using the "pheatmap" package in R.

Candidate target screening

To screen the candidate targets of TRIM56, we construct protein-protein interaction network of TRIM56 interacting proteins and lipid-metabolism-related proteins in NAFLD. The interacting protein information of TRIM56 was obtained from BioGRID database. Lipid-metabolism-related proteins were leading genes in the enriched lipid metabolism related pathways of GSEA. Protein-protein interaction data was download from STRING database. A pair of protein-protein interaction with score >= 400 was considerate as a credible interaction. The protein-protein interaction network was constructed by Cytoscape (version 3.6.0) software. The degree of interacting proteins of Trim56 in network was showed by lollipop chart using the "ggplot" package in R.

Accession number

All RNA-sequencing data support the findings of this study have deposited in NCBI Sequence Read Archive (SRA) with accession number: PRJNA1011487, PRJNA1011905 and PRJNA1011918.

Supplemental Figures

Figure S1

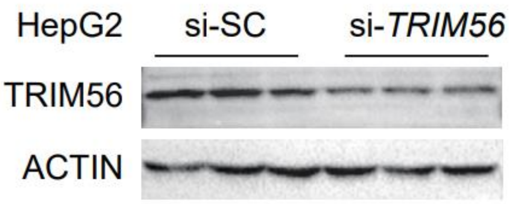

Silencing efficiency of TRIM56 in HepG2 cell. HepG2 cell was transfected with si-*TRIM56* or the scramble control (si-SC) for 48 h before whole cell lysate was collected for western blot, n=3.

Figure S2

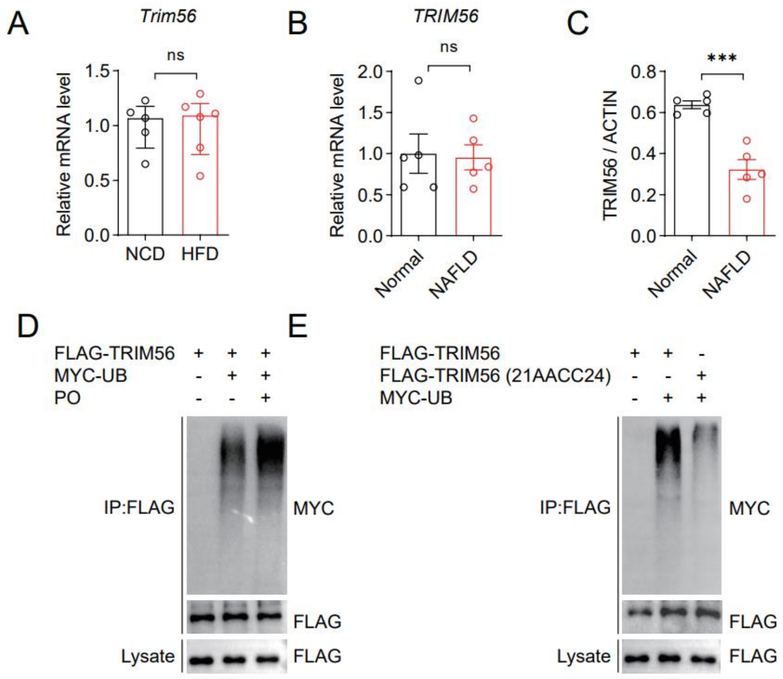

(A) mRNA expression of *Trim56* in the livers from mice fed a normal chow diet (NCD) or high fat diet (HFD) for 24 weeks, n=5 in NCD mice, n=6 in HFD mice. Two-tailed Student's t-test.

(B) mRNA expression of *TRIM56* in the livers from NAFLD patients and the control individuals, n=5. Two-tailed Student's t-test.

(C) The results of densitometric analysis of the western blot shown in Figure 1M, n=5. Two-tailed Student's t-test.

(D) Huh7 were transfected with empty vector (FLAG) or FLAG-TRIM56, or MYC-ubiquitin (UB), in the presence of BSA or PO for 18 h before IP, anti-FLAG

antibody was used for IP to determine ubiquitinated TRIM56, n=3.

(E) HEK293T were transfected with vector (FLAG), FLAG-TRIM56, FLAG-TRIM56 (21AACC24) and MYC-UB, anti-FLAG antibody was used for IP to determine ubiquitinated TRIM56, n=3.

Figure S3

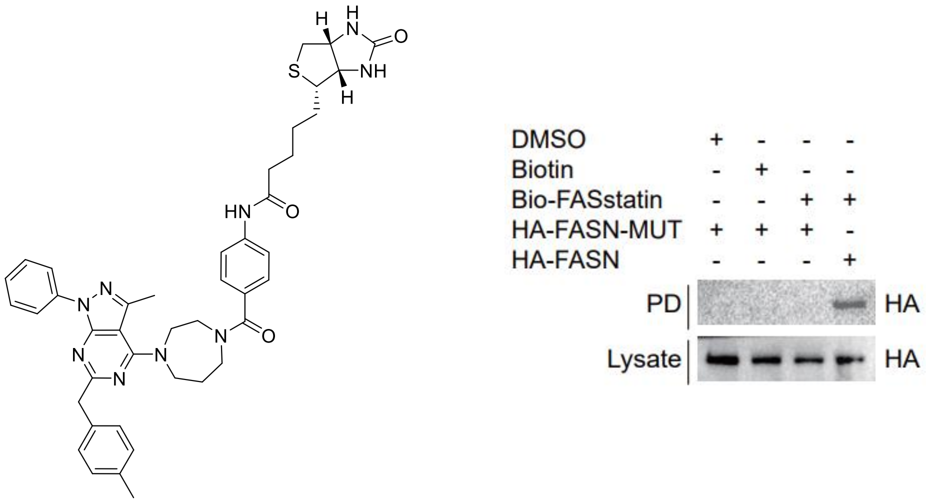

Interaction of biotin-FASstatin with FASN or FASN mutant plasmid in HEK293T. HEK293T cell were transfected with empty vector (HA), HA-FASN and HA-FASN-MUT (2251aa and 2343aa mutant) for 24 h, in the presence of DMSO, biotin or biotin-FASstatin (Bio-FASstatin, chemical structure displayed on the left). Streptavidin-magnetic beads pull down assay was performed followed by western blot analysis, n=3.

Figure S4

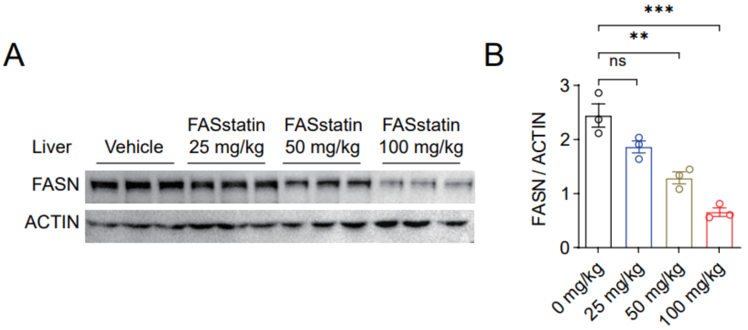

(A) Dose-dependency of FASstatin on hepatic FASN protein expression in mice fed with CDAHFD, n=3. Male C57BL/6J mice were fed with CDAHFD for 2 weeks before treatment with vehicle (0.5% CMC-Na) or three escalating doses of FASstatin (25, 50, 100 mg/kg/d, i.g.) for additional 4 weeks concurrent with feeding with CDAHFD.

(B) The results of densitometric analysis of panel A. n=3. One-way ANOVA followed by the Bonferroni post hoc test.

Figure S5

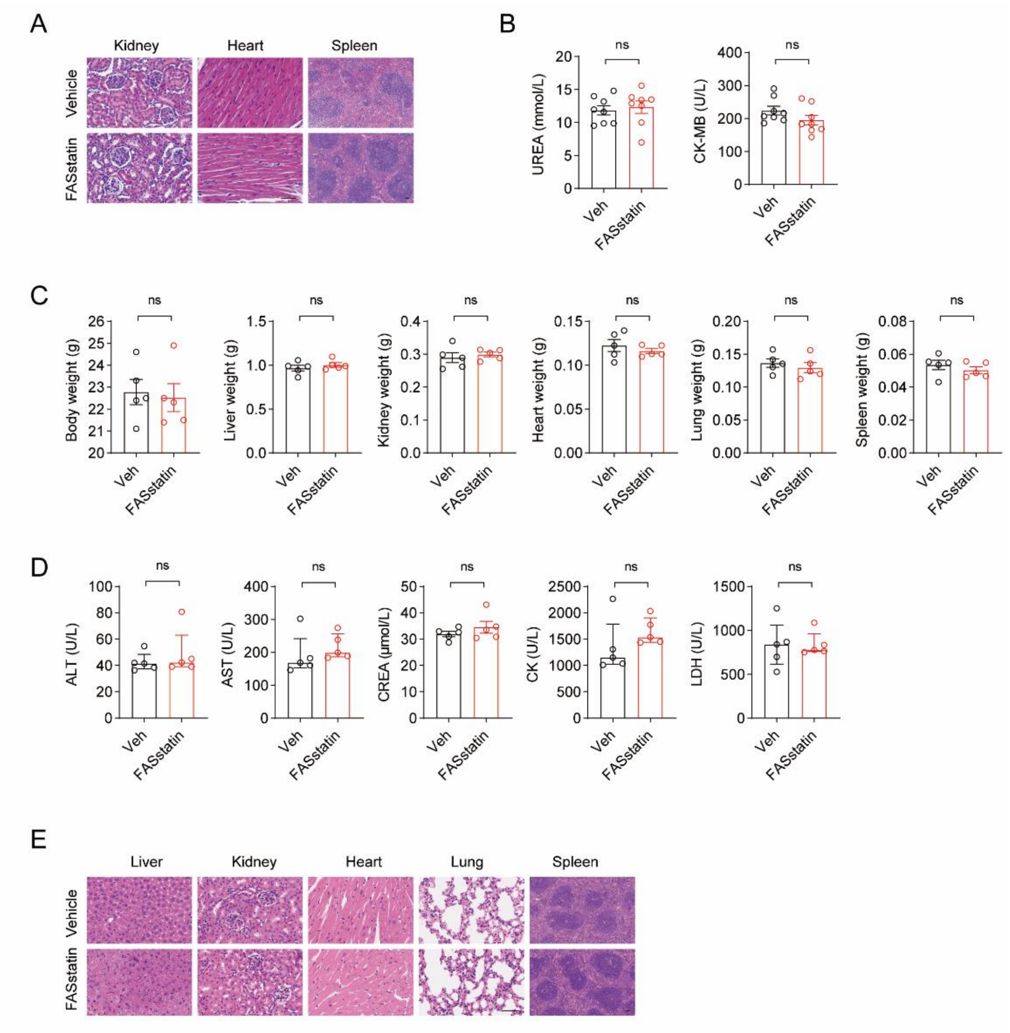

(A) H&E staining of kidney, heart and spleen from mice treated with vehicle or FASstatin (i.g., 50 mg/kg) for 8 weeks under normal chow diet feeding conditions, n=8. Scale bar 50 µm.

(B) Serum levels of UREA and CK-MB were determined in mice treated with vehicle or FASstatin as described in A, n=8. One-way ANOVA followed by the Bonferroni post hoc test.

(C) Acute toxicity experiment. Male C57BL/6J mice were treated with vehicle or FASstatin (i.g., 1750 mg/kg, single dose). 14 days after initial dosing, body weight, liver weight, kidney weight, heart weight, lung weight and spleen weight were determined, n=5. Two-tailed Student's t-test.

(D) Serum levels of ALT, AST, CREA, CK and LDH were determined in mice treated with vehicle or FASstatin (i.g., 1750 mg/kg, single dose), n=5 per group. Mann-Whitney U test for ALT, AST, CK and LDH. Two-tailed Student's t-test for CREA.

(E) H&E staining of liver, kidney, heart, lung and spleen (scale bar 10 μm) from mice treated with vehicle or FASstatin (i.g., 1750 mg/kg, single dose), n=4 per group. Scale bar 50 μm.

Supplemental Tables

Table S1. Primer sequences for real-time PCR.

**Mouse primers**	Sequence (5'-3')
Mouse-*Cd36*-F	CTGGGACCATTGGTGATGAAA
Mouse-*Cd36*-R	CACCACTCCAATCCCAAGTAAG
Mouse-*Scd1*-F	CTGCTGATGTGCTTCATCCT
Mouse-*Scd1*-R	CACCAGAGTGTATCGCAAGAA
Mouse-*Gpam*-F	GAGGAGTCTTCAGTGACAGTTG
Mouse-*Gpam*-R	CAGTCCTCACTGGTGTGTTT
Mouse-*Acsl4*-F	CACACACTTCGACTCACTAGC
Mouse-*Acsl4*-R	GGCTGTCCTTCTTCCCAAAT
Mouse-*Actin*-F	CCGTAAAGACCTCTATGCCAAC
Mouse-*Actin*-R	AGGAGCCAGAGCAGTAATCT
Mouse-*Elovl5*-F	GCGCGGGAGAATCCGATATG
Mouse-*Elovl5*-R	GGTTGTTCTTGCGAAGGATGA
Mouse-*Elovl6*-F	CATGCCGTCATGTACTCTTACT
Mouse-*Elovl6*-R	CATCTGAGTGATCTGGGACAAG
**Human primers**	
Human-*SCD1*-F	TCATAATTCCCGACGTGGCT
Human-*SCD1*-R	CCCAGAAATACCAGGGCACA
Human-*DGAT2*-F	CTGGTTCCCATCTACTCCTTTG
Human- *DGAT2*-R	GGAACTTCTTCTGGACCCATC
Human-*ELOVL6*-F	AACGAGCAAAGTTTGAACTGAGG
Human-*ELOVL6*-R	TCGAAGAGCACCGAATATACTGA
Human-*GPAM*-F	GAAGTCCTGGCTCGTGATTT
Human-*GPAM*-R	TGCTAGTGTGGGTGATTGTG
Human-β-*Actin*-F	CATGTACGTTGCTATCCAGGC
Human-β-*Actin*-R	CTCCTTAATGTCACGCACGAT

F: forward primer   R: reverse primer

Table S2. Key resource table

Reagents	Source	Catalogue number
**Antibodies**		
anti-TRIM56, dil: 1/1000	Abcam	Cat# ab154862
anti-HA, dil:1/1000	Proteintech	Cat# 51064-2-AP
anti-HA, dil:1/1000	Biolight	Cat#TP00973GeA10
anti-Flag, dil:1/1000	MBL	Cat#M185-3L
anti-Flag, dil:1/1000	Biolight	Cat# TP00975GeA10
anti-Flag, dil:1/1000	Proteintech	Cat# 20543-1-AP
anti-myc, dil:1/1000	Proteintech	Cat#10828-1-AP
anti-β-Actin, dil:1/5000	Proteintech	Cat# 23660-1-AP
anti-FASN, dil: 1/1000	IMA	Cat#ASN-108 PA108PR20201201
anti-SCD1, dil: 1/1000	Proteintech	Cat#23393-1-AP
anti-Elovl4, dil: 1/1000	ABclonal	Cat#A3639
anti-Elovl6, dil: 1/1000	Proteintech	Cat# 21160-1-AP
anti-DGAT, dil: 1/1000	Abclonal	Cat# A6610
anti-GPAM, dil: 1/1000	Abclonal	Cat# A6610
**Chemicals, peptides, and recombinant proteins**		
Palmitic acid (PA)	Sigma-Aldrich	CAT#57-10-3
Oleic acid (OA)	Sigma-Aldrich	CAT#01008-5-G
Fatty-acid free BSA	Equitech Bio	Cat#BAH66-0100
MG132	TargetMol	Cat#133407-82-6
Chloroquine (CQ)	Sigma-Aldrich	Cat#C6628
C75	MedChemExpress	Cat#HY-12364
C75	Selleck Chemicals	Cat#S9819
Lipopolysaccharide (LPS)	Sigma-Aldrich	Cat#L4391
TGFβ1	PeproTech	Cat#100-21-10
DMSO	Sigma-Aldrich	Cat#P3761
Protein G Agarose beads	Millipore	Cat#16-266
Opti-MEM	Thermo Fisher	Cat#31985062
Lipo2000	Thermo Fisher	Cat# L3000015
Nile Red	Invitrogen	Cat#n1142
BODIPY	Invitrogen	Cat#d3922
RIPA buffer	Shanghai Sangon	Cat#C500005-0100
TRIzol	Sigma-Aldrich	Cat#T9424
DAPI	Invitrogen	Cat#S36939
Hochest	Invitrogen	Cat#H3570

SYBR Green Master Mix	Vazyme	Cat# Q121-02
PhosStop phosphatase inhibitor	Roche	Cat#4906837001
Complete protease inhibitor cocktail	Sigma-Aldrich	Cat#P8340
Clarity Western ECL Substrate	Bio-Rad	Cat#1705061
Dulbecco’s modified Eagle’s medium (DMEM)	GIBCO	Cat# C11995500BT
fetal bovine serum (FBS)	Tico Europe	Cat#FBSEU500
Polybrene	Sigma	Cat#H9268
Puromycin	GIBCO	Cat#A1113803
Trypsin	GIBCO	Cat#27250-018
1% penicillin/streptomycin	GIBCO	Cat#15140-122
Sudan III	Solarbio	Cat#S8460
Hematoxylin	Service Bio	Cat#G1004
Eosin	BASO	Cat#BA-4024
Tissue-Tek O.C.T. Compound	Service Bio	Cat#4583
Oil Red O	Sigma-Aldrich	Cat# 625
Liver Perfusion Medium	Thermo Fisher Scientific	Cat#17701-038
Liver Digestion Medium	Thermo Fisher Scientific	Cat#17701-034
Pronase E	Roche	Cat#10165921001
Collagenase IV	GIBCO	Cat#17104019
DNase I	Roche	Cat#10104159001
Crystal violet	Sinopharm Chemical Reagent	Cat#71012314
BCA Protein assay kit	Thermo Fisher Scientific	Cat#23225
Transcriptor first-strand cDNA synthesis kit	Vazyme	Cat# R212-01
Triglyceride (TG) assay kit	Wako	Cat#290-63701
Cholesterol assay kit	Wako	Cat#294-65801
Cell Counting Kit-8 assay kit	Shanghai Sangon	Cat# E606335-0100
RNA kit	Shanghai Yishan	Cat# RN001
RNA 6000 Nano kit	Agilent	Cat#5067-1511
MGIEasy RNA Library Prep Kit	MGI Tech Co.	Cat#1000006384
TRIM family siRNA library	Ribobio	Cat# STLC002

Scheme 1: Chemical synthesis of FASstatin (V012-3807, FASNi#8)

<u>1. Synthesis route of FASstatin (V012-3807)</u>

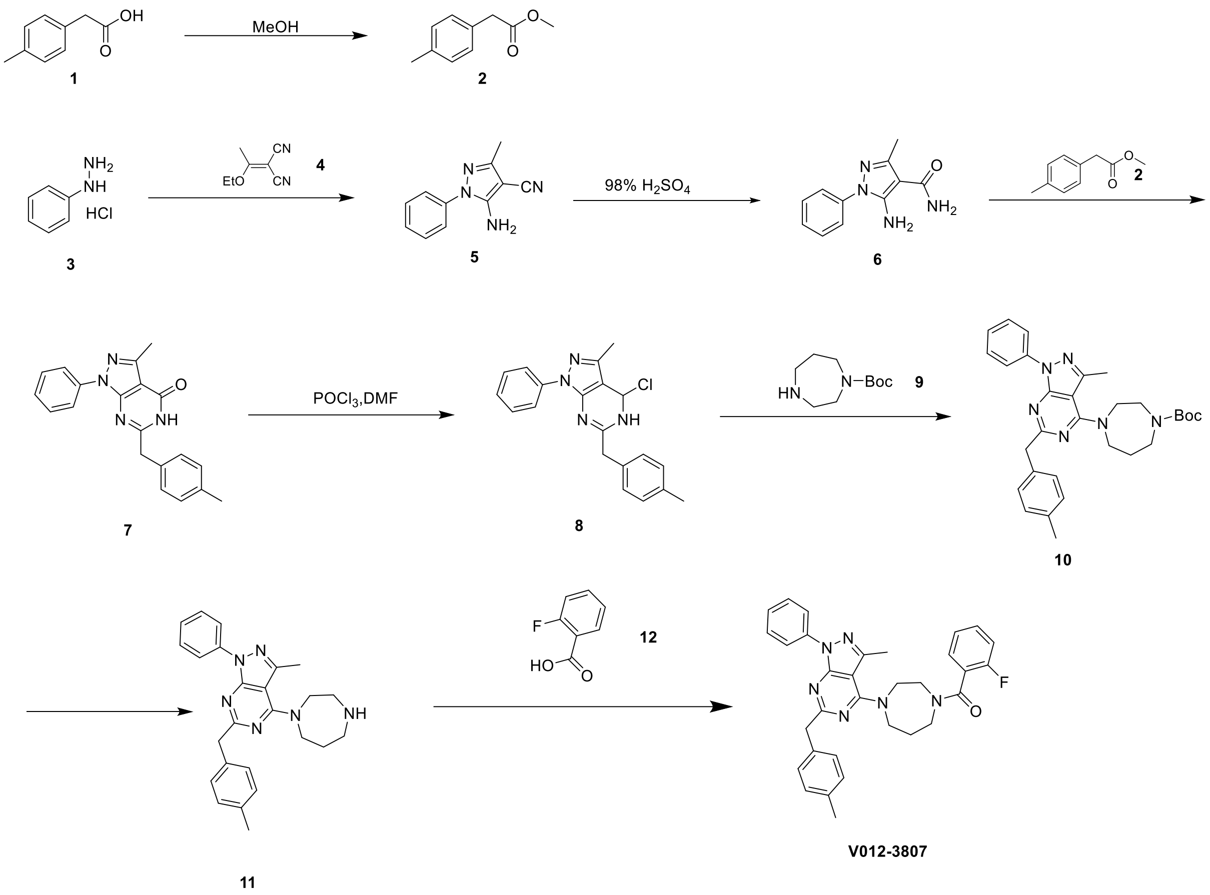

2. Detailed synthesis procedures of FASstatin (V012-3807)

Synthesis of Compound 5

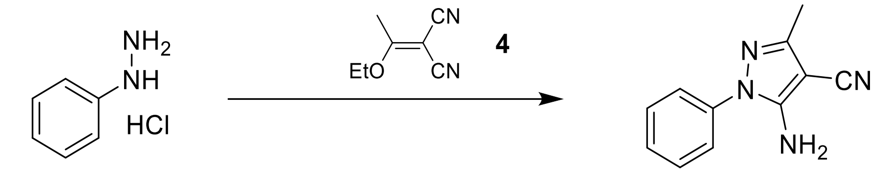

3 5

To a mixture of **Compound 3** (10 g, 1 eq) in EtOH (400 mL) was added **Compound 4** (9.42 g, 1 eq) and NaOAc (9.4 g, 1 eq). The reaction mixture was stirred at 85 °C for 4 h. The mixture was poured into water (300 mL), extracted with EA (300 mL x 3), washed with brine (250 mL x 2), concentrated under vacuum to give a residue, **Compound 5** (crude 15.2 g) was obtained as red solid.

Synthesis of Compound 2

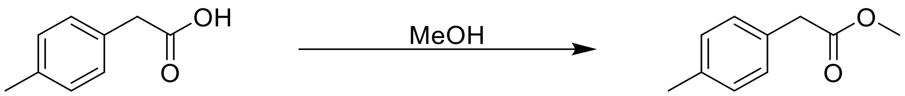

1 2

To a mixture of **Compound 1** (40 g, 1 eq) in MeOH (400 mL) was added **98% H_2SO_4** (0.5ml). The mixture was stirred at 65 °C for 12 h. The mixture solvent removed in vacuo, poured into $NaHCO_3$ (200 mL), extracted with EA (200 mL x 2), washed with brine (250 mL x 2), concentrated under high vacuum to give residue. **Compound 2** (crude 45 g) was obtained as colorless oil.

Synthesis of Compound 6

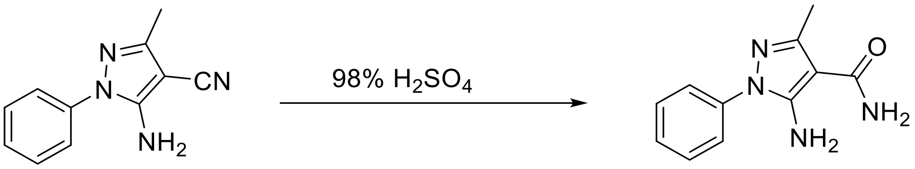

5 6

Compound 5 (15. 2 g, 1 eq) was dissolved in 98%H_2SO_4 (80 mL). The mixture was stirred at 90 °C for 2 h. The reaction was then quenched by the addition to water/ice (200 mL). The pH value of the solution was adjusted to about 8-9 with potassium carbonate. The solid was collected by filtration, dried. **Compound 6** (11.4 g, 70.02% yield) was obtained as red solid.

Synthesis of Compound 7

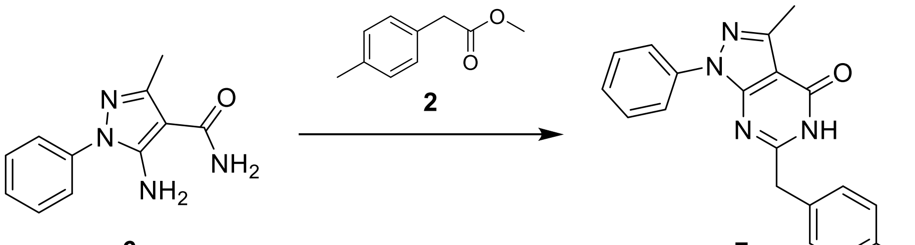

6 7

To a mixture of **Compound 6** (11.4 g, 1 eq) and **Compound 2** (26g, 3eq) in EtOH (200 mL) was added sodium ethoxide (21.52g, 6 eq) which dissolved in EtOH(300ml) and stirred at 85 °C for 12 h. The mixture was poured into HOAc (10%, 50mL). The solid was collected by filtration, **Compound 7**(15 g, 86.15% yield) was obtained as white solid.

Synthesis of Compound 8

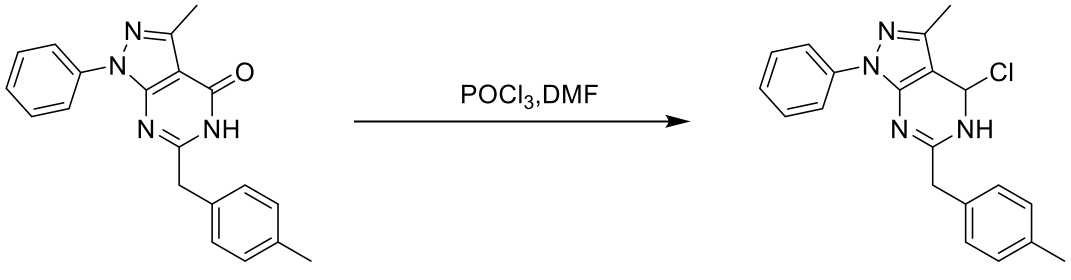

7 **8**

To a mixture of **Compound 7** (4.5 g, 1 eq) in $POCl_3$ (25 mL) was added DMF (1 drop). The mixture was stirred at 110 °C for 2 h. The mixture was poured into ice-water slowly, extracted with EA (50mL x3), washed with brine(100mL), concentrated under high vacuum to give the residue. **Compound 8** (4.2 g, crude) was obtained as light yellow solid.

Synthesis of Compound 10

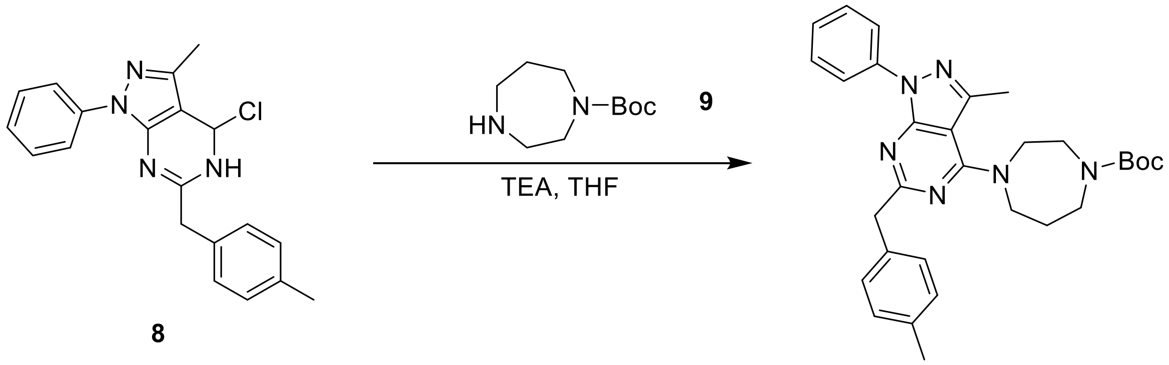

8 **10**

To a mixture of **Compound 8** (4.2 g, 1 eq) in THF (150 mL) was added **Compound 9**(2.88g 1.2eq) and TEA (3.64g, 3eq). The mixture was stirred at 50 °C for 12 h. The mixture was poured into water(150mL), extracted with EA(150mL x3),washed with brine(100mL x2),concentrated under high vacuum to give the residue. The residue was purified by flash silica gel column (PE:EA=10:0 to 83:17) , **Compound 10** (6.05 g, 98.53% yield) was obtained as white solid.

Synthesis of Compound 11

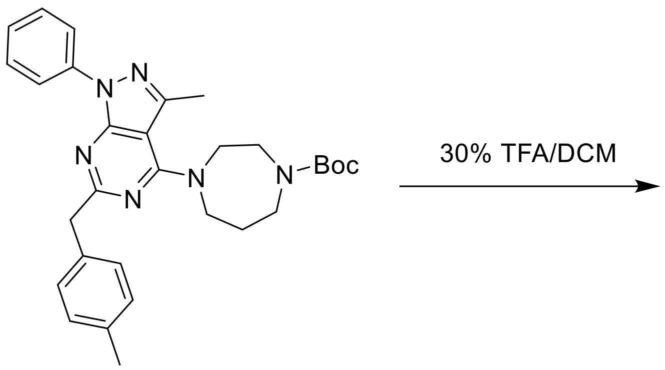

10

11

To a mixture of **Compound 10** (6.05 g, 1 eq) in DCM (96 mL) was added **TFA** (24mL). The mixture was stirred at 25 °C for 4 h. The mixture was concentrated under high vacuum to give residue, diluted with EA (20mL), the product was filtered off, washed with water, and dried. **Compound 11** (5.5g, 99.07%yield) as yellow solid.

Synthesis of Compound 11

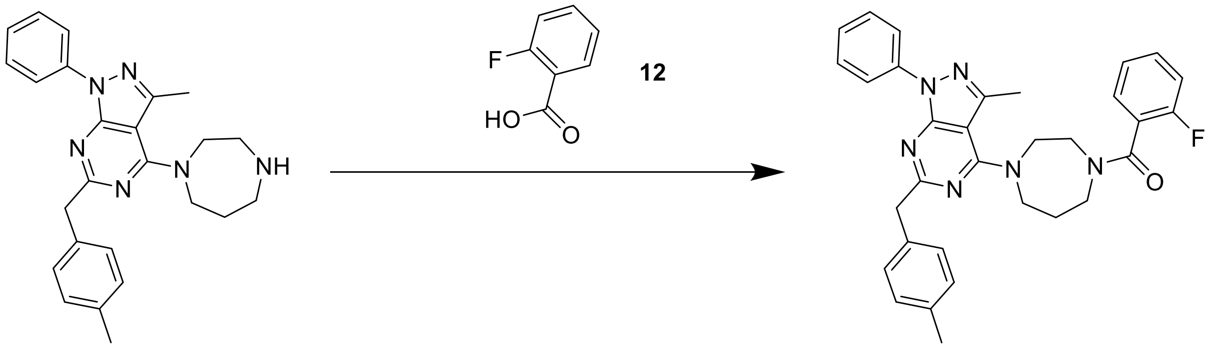

11 V012-3807

To a mixture of **Compound 12** (1.87 g, 1 eq) in DMF (96 mL) was added **HATU** (5.58g, 1.1eq), DIPEA (5.17g, 3eq). The mixture was stirred at 25 °C for 0.5 h, followed by **Compound 11**(5.5g, 1eq). The mixture was poured into water(100mL), extracted with EA (80mLx3), washed with water(100mLx3), concentrated under high vacuum to give residue, The residue was purified by flash silica gel column (PE:EA=10:0 to1:1). **V012-3807** (5.09g, 98.57%yield) was obtained as brown solid.

LC-MS, $[M+H]^+$=535.4

^{1}H NMR (400 MHz, $CDCl_3$) δ 8.19 (dd, *J* = 8.0, 2.9 Hz, 2H), 7.54 – 7.48 (m, 2H), 7.46 – 7.38 (m, 1H), 7.32 (dd, *J* = 11.0, 5.7 Hz, 2H), 7.21 – 7.05 (m, 6H), 4.14 – 3.67 (m, 8H), 3.57 (s, 1H), 3.35 (t, *J* = 6.2 Hz, 1H), 2.70 (d, *J* = 13.5 Hz, 3H), 2.32 (d, *J* = 7.3 Hz, 3H), 2.14 (s, 1H), 1.94 – 1.82 (m, 1H).

References:

1. Yang JD, Abdelmalek MF, Pang H, et al. Gender and menopause impact severity of fibrosis among patients with nonalcoholic steatohepatitis. Hepatology 2014;59:1406-14.
2. Li Y, Xu J, Lu Y, et al. DRAK2 aggravates nonalcoholic fatty liver disease progression through SRSF6-associated RNA alternative splicing. Cell Metab 2021;33:2004-2020.e9.
3. Zhao P, Sun X, Chaggan C, et al. An AMPK-caspase-6 axis controls liver damage in nonalcoholic steatohepatitis. Science 2020;367:652-660.
4. Wang L, Zhang X, Lin ZB, et al. Tripartite motif 16 ameliorates nonalcoholic steatohepatitis by promoting the degradation of phospho-TAK1. Cell Metab 2021;33:1372-1388.e7.
5. Kodama T, Yi J, Newberg JY, et al. Molecular profiling of nonalcoholic fatty liver disease-associated hepatocellular carcinoma using SB transposon mutagenesis. Proc Natl Acad Sci U S A 2018;115:E10417-e10426.
6. Moon SH, Huang CH, Houlihan SL, et al. p53 Represses the Mevalonate Pathway to Mediate Tumor Suppression. Cell 2019;176:564-580.e19.
7. Zhang XJ, Ji YX, Cheng X, et al. A small molecule targeting ALOX12-ACC1 ameliorates nonalcoholic steatohepatitis in mice and macaques. Sci Transl Med 2021;13:eabg8116.
8. Trott O, Olson AJ. AutoDock Vina: improving the speed and accuracy of docking with a new scoring function, efficient optimization, and multithreading. J Comput Chem 2010;31:455-61.
9. Wang Z, Wang M, Zhang M, et al. High-affinity SOAT1 ligands remodeled cholesterol metabolism program to inhibit tumor growth. BMC Med 2022;20:292.
10. Wang Z, Wu W, Guan X, et al. 20(S)-Protopanaxatriol promotes the binding of P53 and DNA to regulate the antitumor network via multiomic analysis. Acta Pharm Sin B 2020;10:1020-1035.
11. Xu S, Xu Y, Liu P, et al. The novel coronary artery disease risk gene JCAD/KIAA1462 promotes endothelial dysfunction and atherosclerosis. Eur Heart J 2019;40:2398-2408.
