# Supplementary material for: TRIM56 protects against nonalcoholic fatty liver disease by promoting the degradation of fatty acid synthase
Source: J Clin Invest. 2024 Jan 11;134(5):e166149. doi: 10.1172/JCI166149 (PMC10904058; doi:10.1172/JCI166149)

Uncropped gels for Western Blots in Figure 1

F1-H

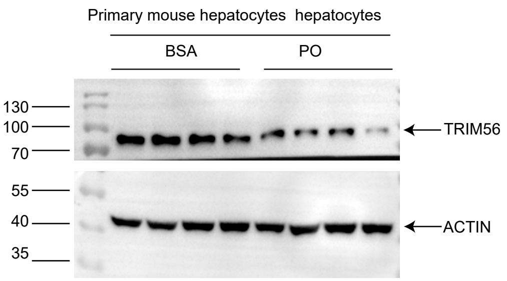

F1-I

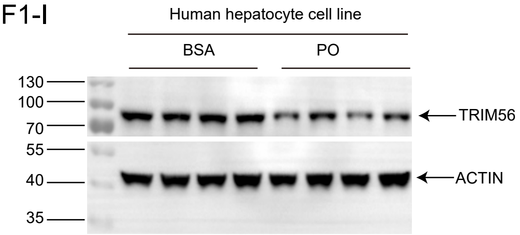

F1-J

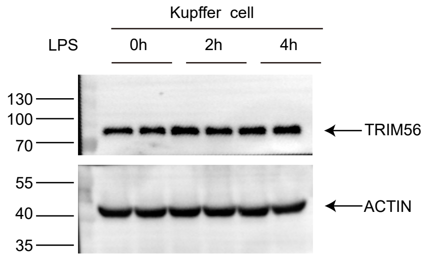

F1-K

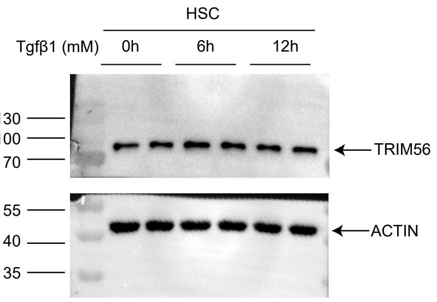

F1-L

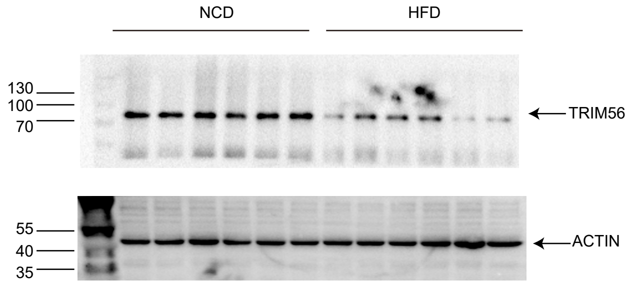

F1-M

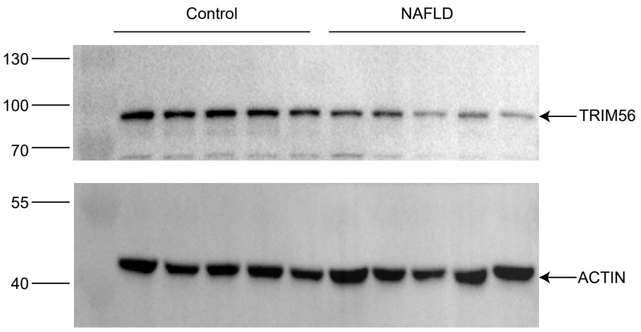

Uncropped gels for Western Blots in Figure 2

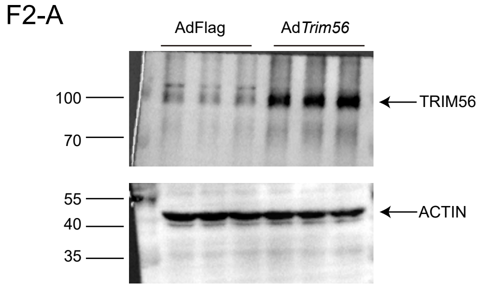

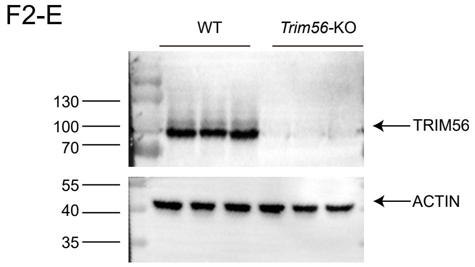

Uncropped gels for Western Blots in Figure 3

F3-A

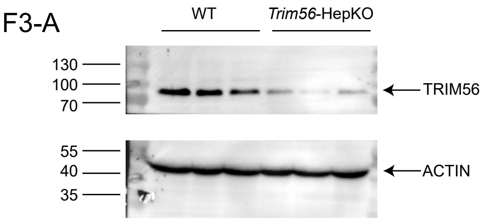

F3-K

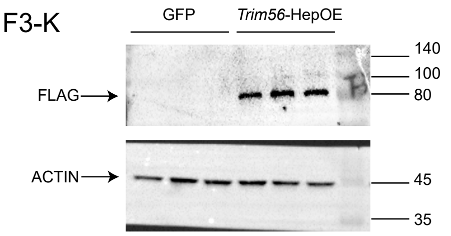

Uncropped gels for Western Blots in Figure 4

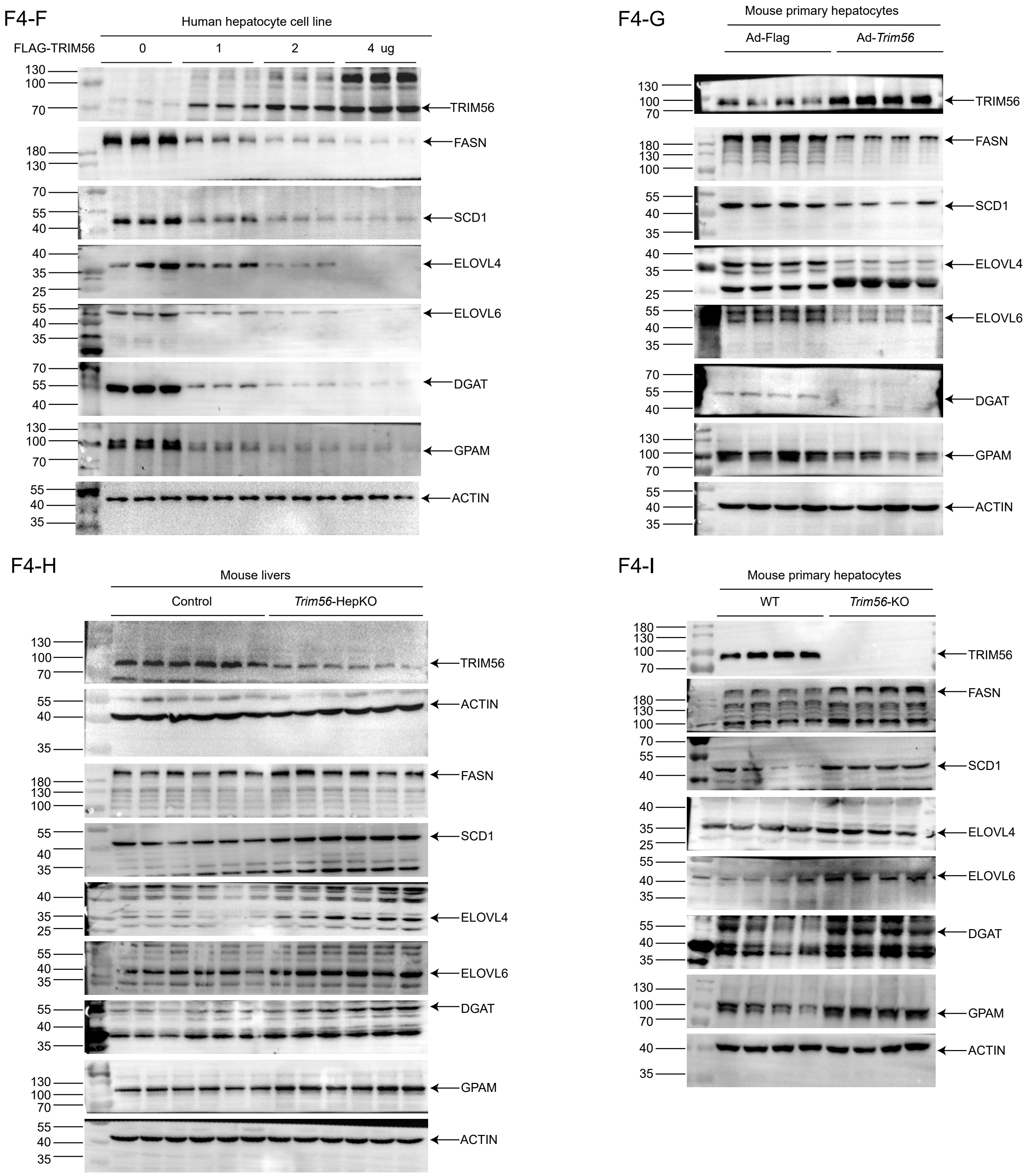

Uncropped gels for Western Blots in Figure 5

F5-A

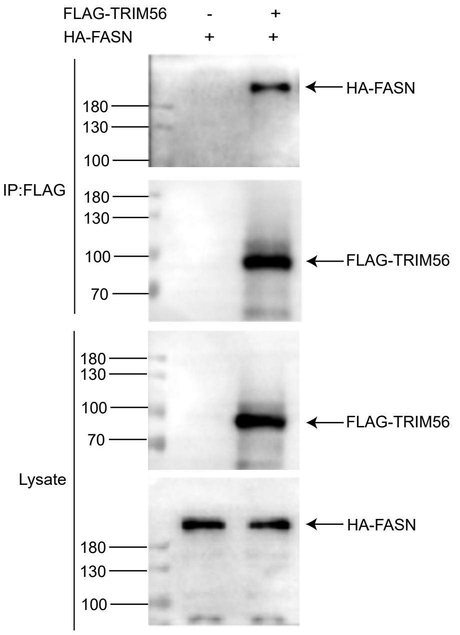

F5-B

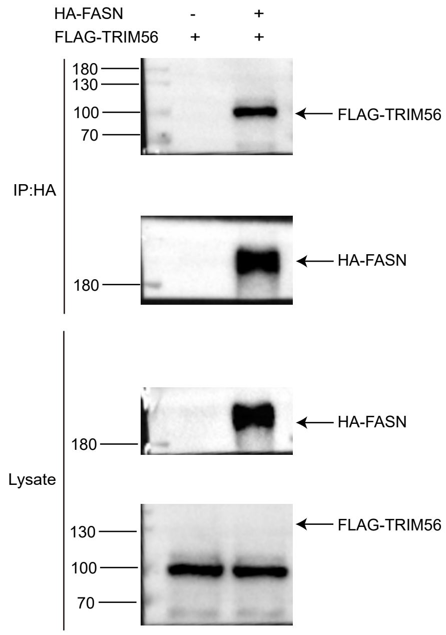

F5-C

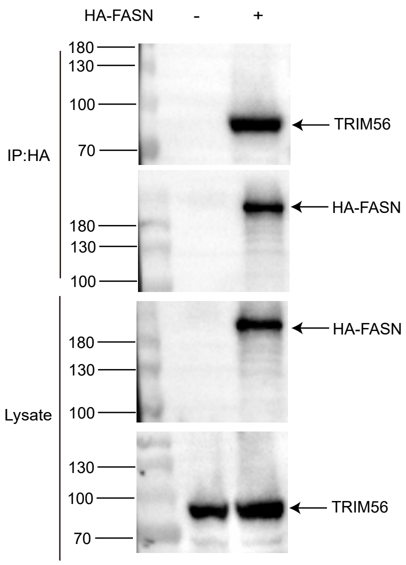

F5-D

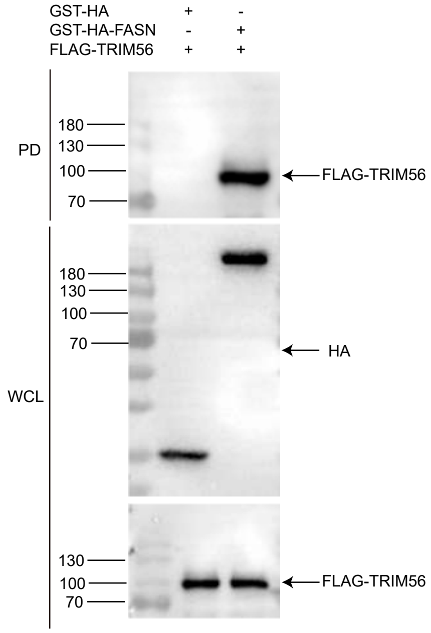

F5-E

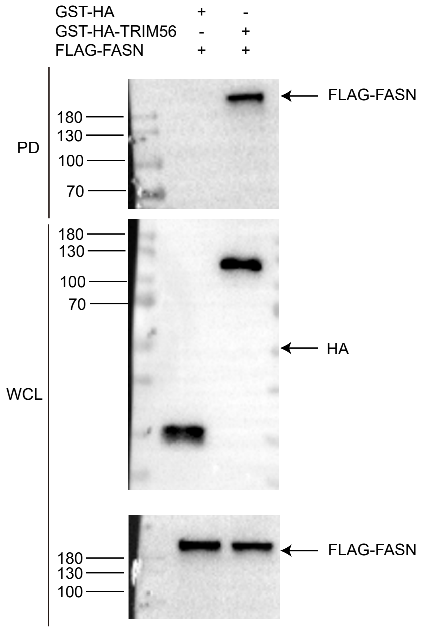

F5-G

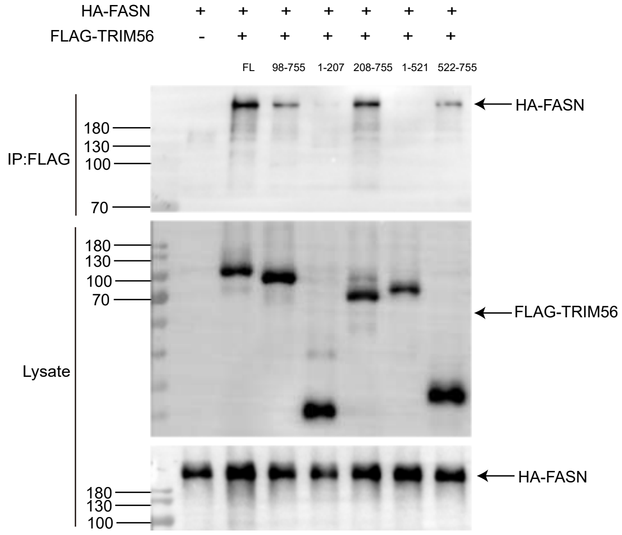

F5-J

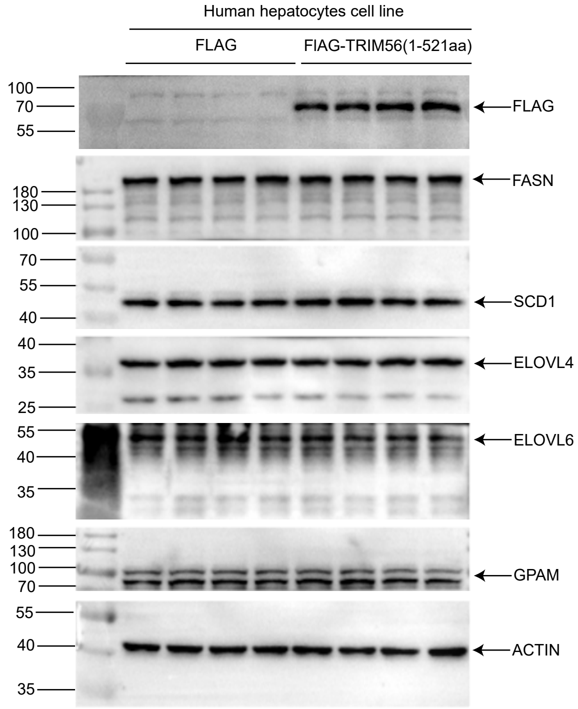

Uncropped gels for Western Blots in Figure 6

F6-A

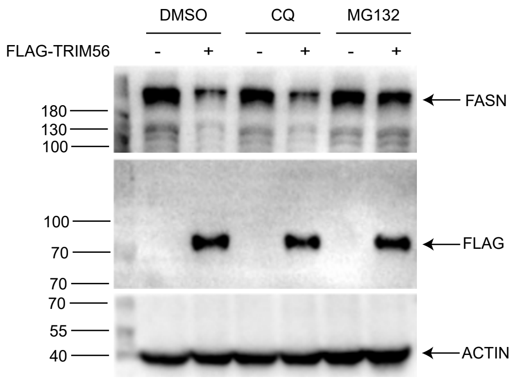

F6-B

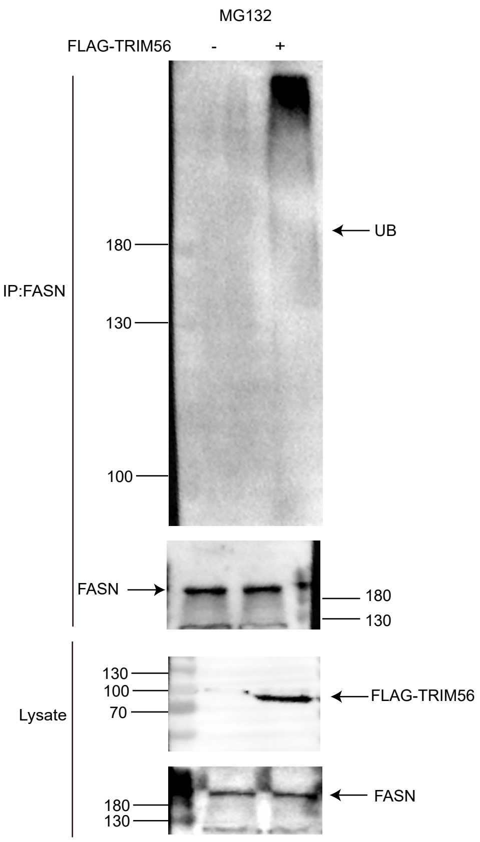

F6-C

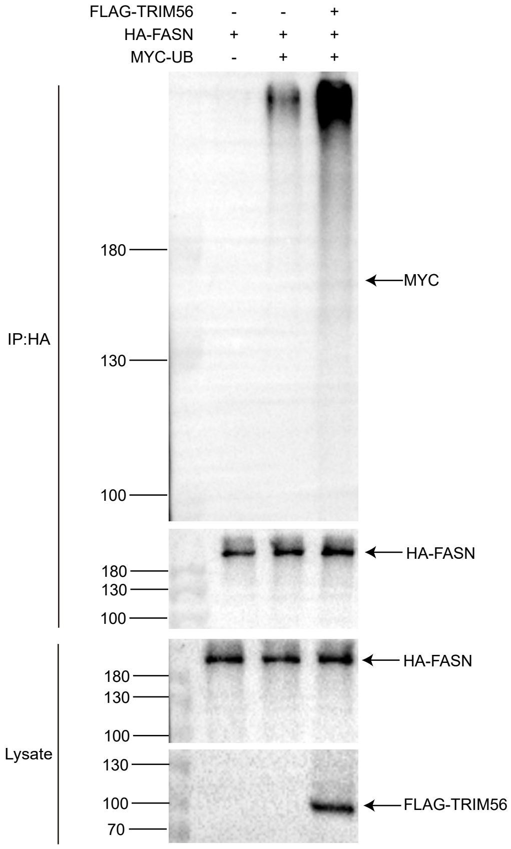

F6-D

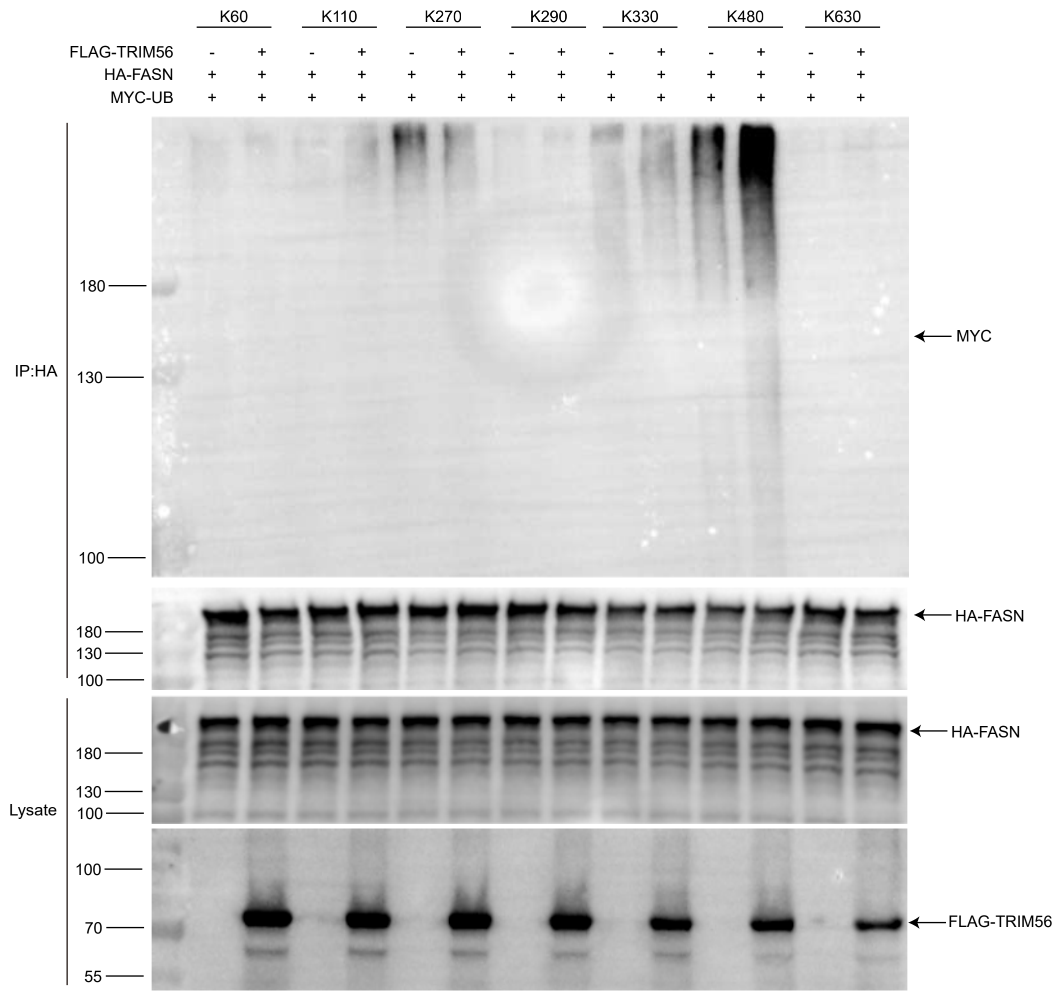

Uncropped gels for Western Blots in Figure 6

F6-E

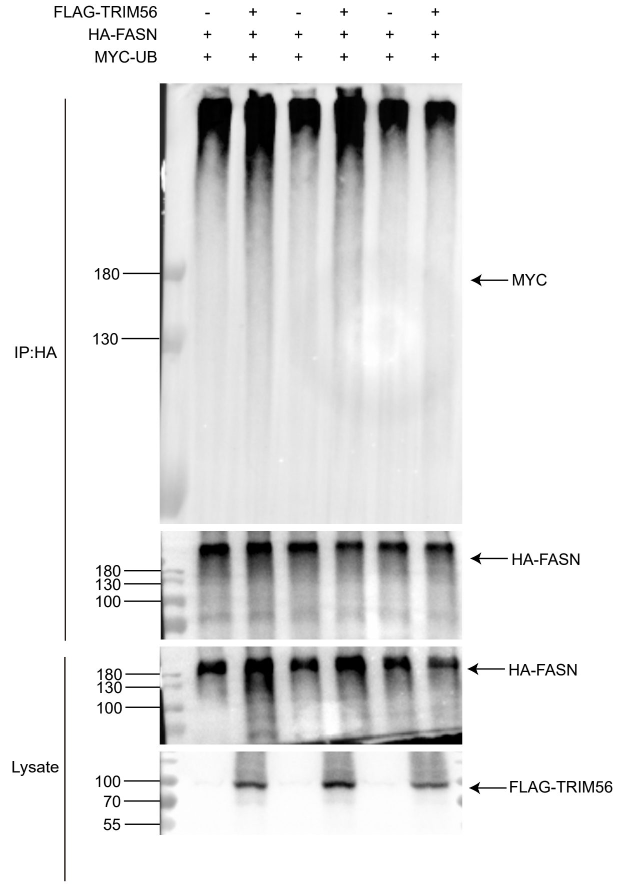

F6-F

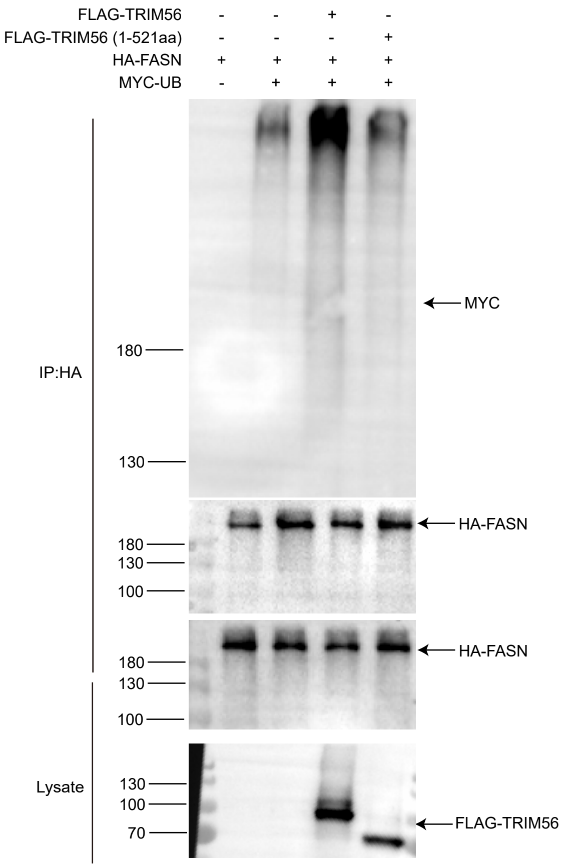

F6-G

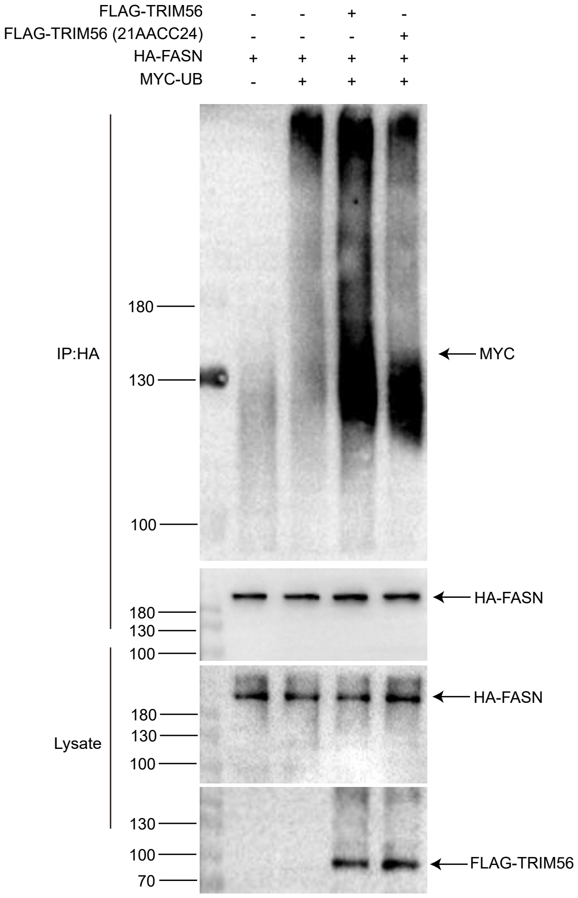

F6-J

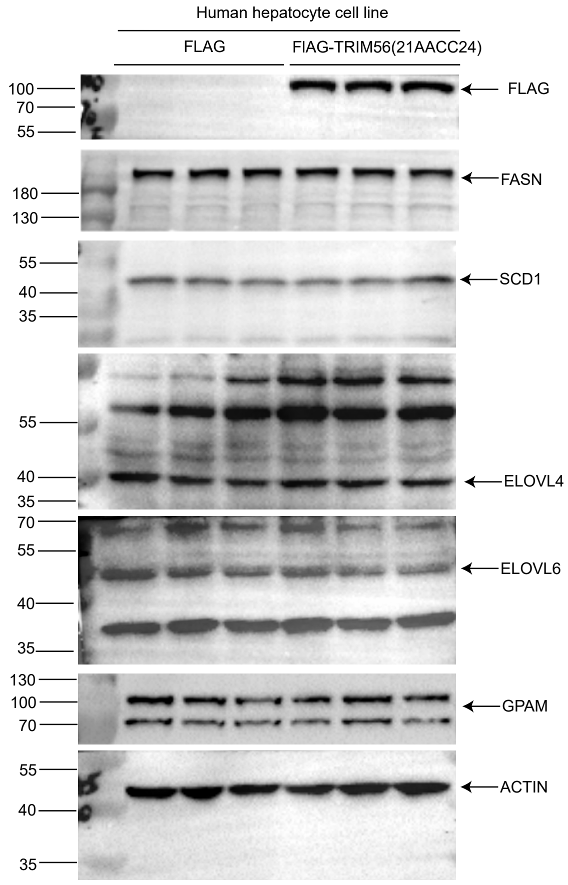

Uncropped gels for Western Blots in Figure 7

F7-A

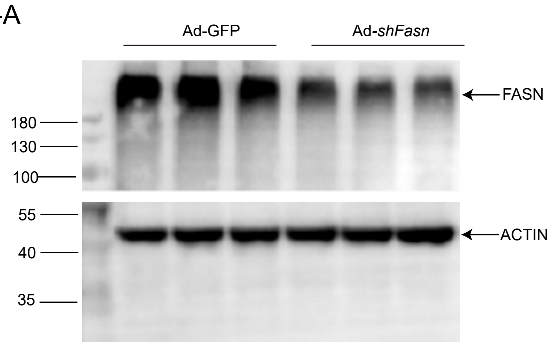

F7-E

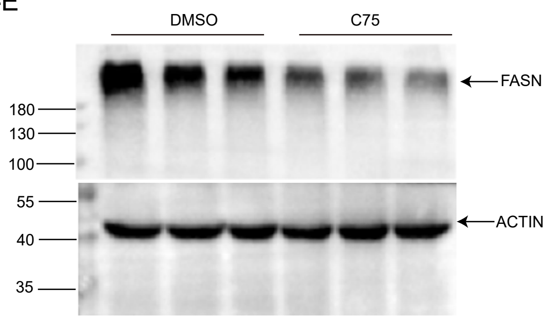

Uncropped gels for Western Blots in Figure 8

F8-F

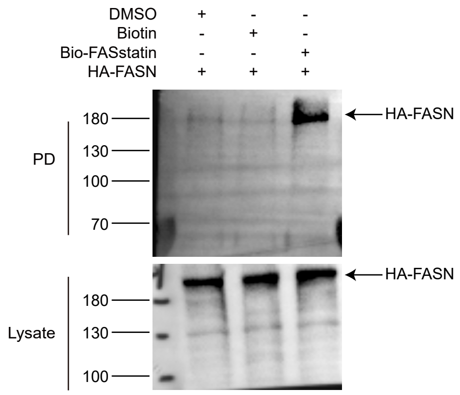

F8-H

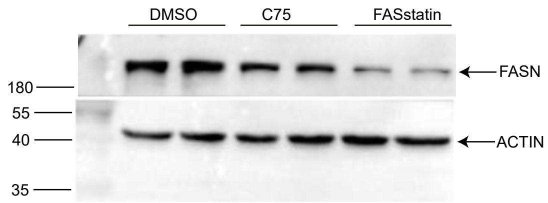

F8-L

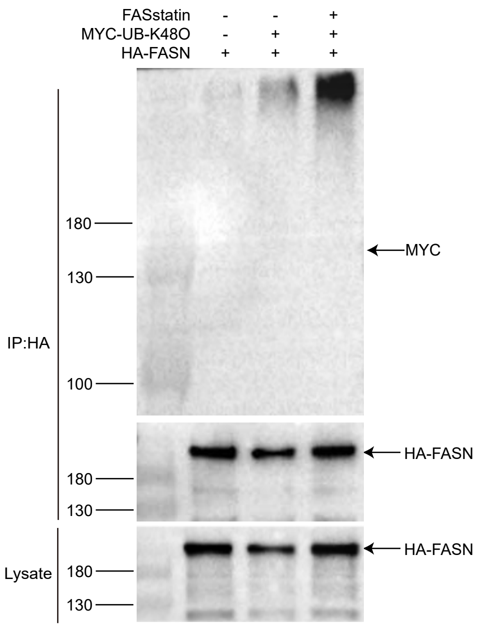

F8-M

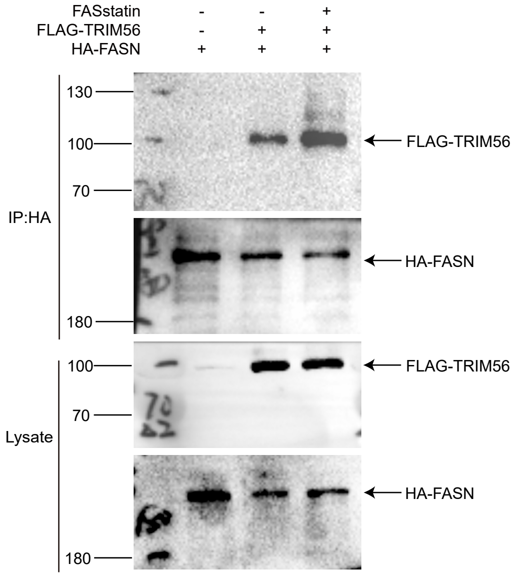

F8-N

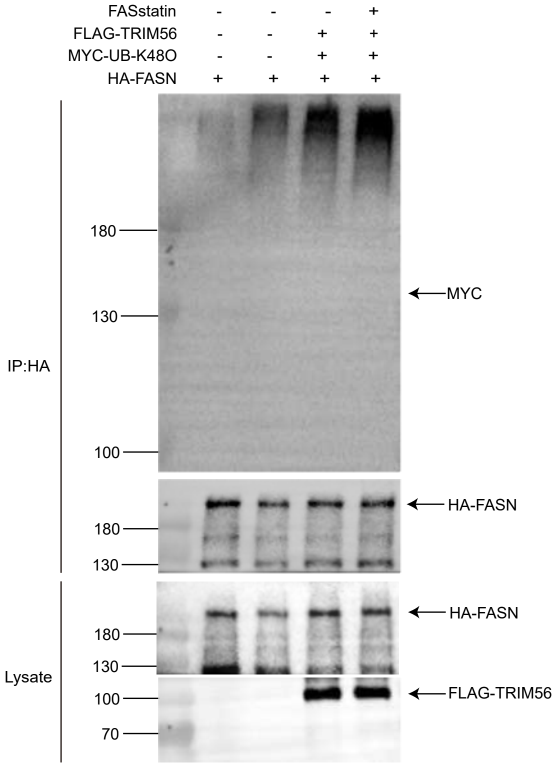

Uncropped gels for Western Blots in Figure 9

F9-A

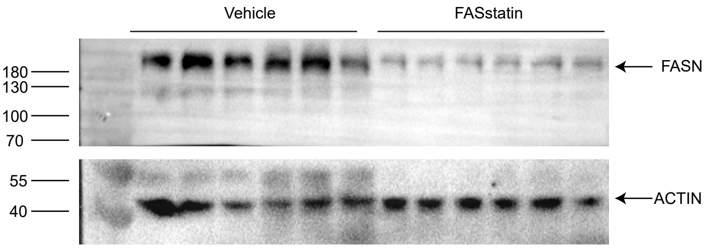

F9-B

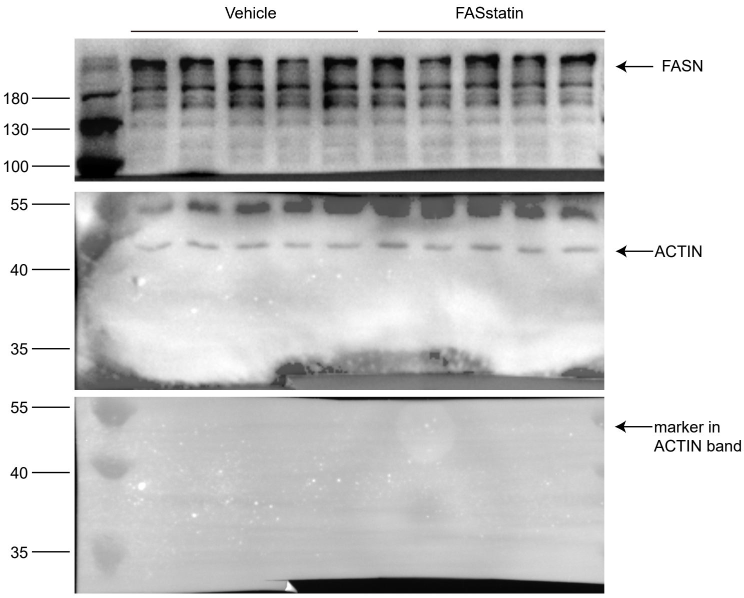

F9-L

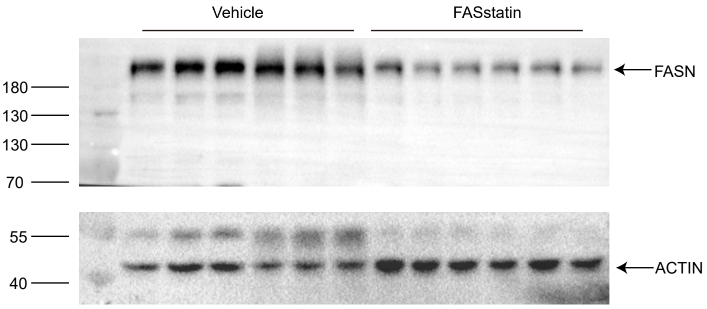

Uncropped gels for Western Blots in Suppl.Figure

Suppl.Figure 1

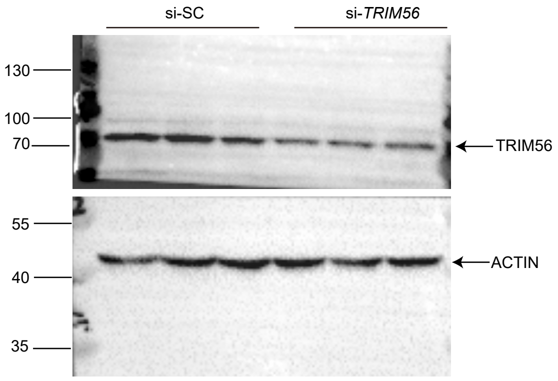

Suppl.Figure 2

D

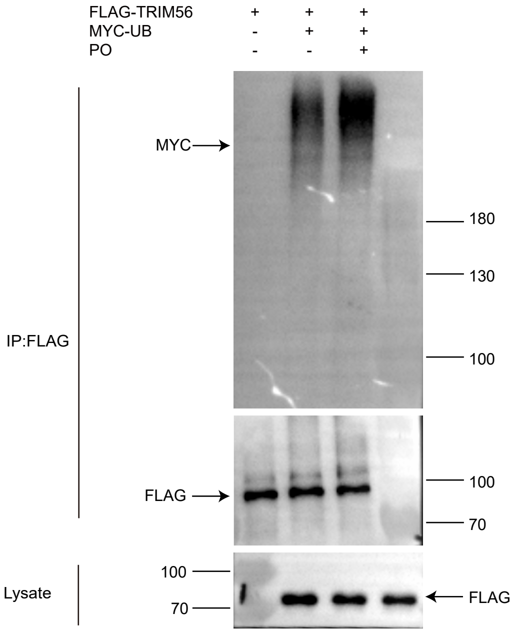

E

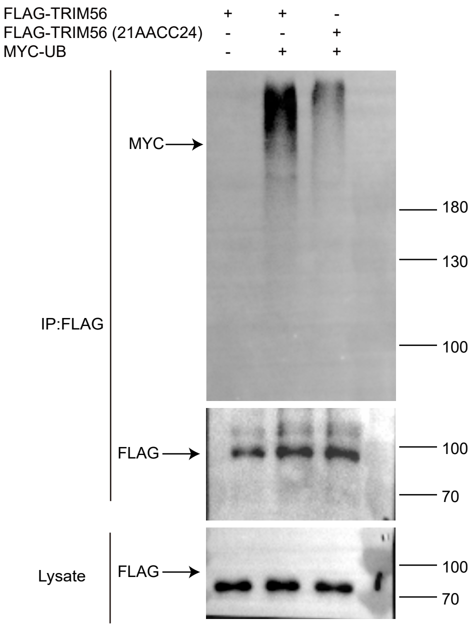

Suppl.Figure 3

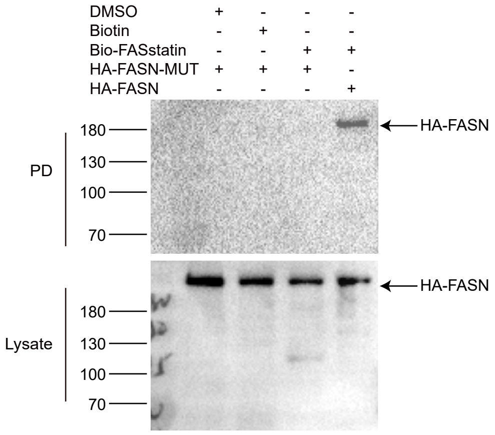

Suppl.Figure 4

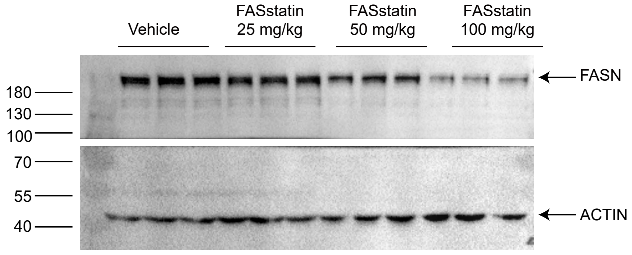

Supplement: Unedited blot and gel images [file jci-134-166149-s277.pdf]
